# The interleukin-4/PPARγ signaling axis promotes oligodendrocyte differentiation and remyelination after brain injury

**DOI:** 10.1371/journal.pbio.3000330

**Published:** 2019-06-21

**Authors:** Qingxiu Zhang, Wen Zhu, Fei Xu, Xuejiao Dai, Ligen Shi, Wei Cai, Hongfeng Mu, T. Kevin Hitchens, Lesley M. Foley, Xiangrong Liu, Fang Yu, Jie Chen, Yejie Shi, Rehana K. Leak, Yanqin Gao, Jun Chen, Xiaoming Hu

**Affiliations:** 1 Pittsburgh Institute of Brain Disorders and Recovery, and Department of Neurology, School of Medicine, University of Pittsburgh, Pittsburgh, Pennsylvania, United States of America; 2 Geriatric Research, Education and Clinical Center, Veterans Affairs Pittsburgh Health Care System, Pittsburgh, Pennsylvania, United States of America; 3 Animal Imaging Center, School of Medicine, University of Pittsburgh, Pittsburgh, Pennsylvania, United States of America; 4 Department of Neurobiology, School of Medicine, University of Pittsburgh, Pittsburgh, Pennsylvania, United States of America; 5 Graduate School of Pharmaceutical Sciences, Duquesne University, Pittsburgh, Pennsylvania, United States of America; UCSD, UNITED STATES

## Abstract

The repair of white matter damage is of paramount importance for functional recovery after brain injuries. Here, we report that interleukin-4 (IL-4) promotes oligodendrocyte regeneration and remyelination. IL-4 receptor expression was detected in a variety of glial cells after ischemic brain injury, including oligodendrocyte lineage cells. IL-4 deficiency in knockout mice resulted in greater deterioration of white matter over 14 d after stroke. Consistent with these findings, intranasal delivery of IL-4 nanoparticles after stroke improved white matter integrity and attenuated long-term sensorimotor and cognitive deficits in wild-type mice, as revealed by histological immunostaining, electron microscopy, diffusion tensor imaging, and electrophysiology. The selective effect of IL-4 on remyelination was verified in an ex vivo organotypic model of demyelination. By leveraging primary oligodendrocyte progenitor cells (OPCs), microglia-depleted mice, and conditional OPC-specific peroxisome proliferator-activated receptor gamma (PPARγ) knockout mice, we discovered a direct salutary effect of IL-4 on oligodendrocyte differentiation that was mediated by the PPARγ axis. Our findings reveal a new regenerative role of IL-4 in the central nervous system (CNS), which lies beyond its known immunoregulatory functions on microglia/macrophages or peripheral lymphocytes. Therefore, intranasal IL-4 delivery may represent a novel therapeutic strategy to improve white matter integrity in stroke and other brain injuries.

## Abstract 2

The repair and remyelination of white matter are of paramount importance for functional recovery after brain injuries. This study shows that interleukin-4 plays an essential role in oligodendrocyte differentiation and long-term white matter recovery, beyond its well-known immunoregulatory functions, and is mediated by the PPARγ axis.

## Introduction

The primary focus of stroke research has traditionally been on neuronal damage within gray matter, but emerging evidence reveals a robust correlation between white matter injury and long-term sensorimotor and cognitive deficits after stroke [1]. White matter accounts for approximately 60% of total brain volume in humans. It is composed mostly of bundled axons and glial cells, including myelin-producing oligodendrocytes, astrocytes, and microglia. Large axons in the brain are enwrapped by the insulating myelin sheath, which greatly accelerates neural transmission along nerve fibers. White matter damage after stroke involves multiple mechanisms, including demyelination, degeneration of axons, and neuroinflammation [2–4]. The vulnerability of oligodendrocytes to ischemic injury and the resultant demyelination have been reported in numerous publications using different stroke models [5–8]. Remyelination is a process whereby new myelin sheaths are generated around demyelinated axons in the adult central nervous system (CNS) after injuries. It is one of the critical regenerative mechanisms for white matter repair after brain injuries. The differentiation of oligodendrocyte progenitor cells (OPCs) into mature oligodendrocytes plays a central role in remyelination. OPCs are present in the normal adult brain [9]. Both the proliferation and migration of OPCs appear to increase in the adult ischemic brain [10], suggestive of endogenous efforts at remyelination and white matter repair. Cellular fate mapping studies have demonstrated the differentiation of OPCs toward the oligodendrocyte lineage in the ischemic brain, but the majority of these cells fail to develop into mature, functional oligodendrocytes [11–13]. As a result of this failure, white matter integrity after stroke shows little improvement over time [10]. Aside from these roadblocks, the efficacy of oligodendrocyte regeneration and axon remyelination also declines with age [14]. Without proper remyelination, naked axons are degraded, thereby interrupting neuronal communication and leading to functional disabilities. Therefore, therapeutic interventions to promote the differentiation of OPCs into mature oligodendrocytes and enhance the remyelination of axons are promising strategies to achieve successful white matter repair and functional recovery after stroke [11].

Interleukin-4 (IL-4) is an anti-inflammatory cytokine produced by a variety of immune cells, but accumulating evidence indicates that IL-4 harbors functions beyond immune modulation in the CNS. For example, one early report suggested that IL-4 promoted learning and memory capacities in the normal brain [15]. This conclusion, however, was challenged by more recent studies demonstrating equivalent baseline cognitive functions between wild-type (WT) and IL-4 knockout (KO) genotypes [16, 17]. Instead, the IL-4 KO mice were shown to display anxiety-like behavior [17]. Despite these discrepancies in studies of IL-4 KO mice, the importance of IL-4 in maintaining or restoring neurological functions under pathological conditions is widely accepted. For example, the reduction of IL-4 levels with advanced age is thought to contribute to the cognitive decline in aged populations and in Alzheimer disease [18]. We previously documented increased expression of IL-4 and the IL-4 receptor (IL-4Rα) in the ischemic brain [16]. IL-4 may be released from injured neurons and peripheral immune cells that have infiltrated the brain after stroke [19]. IL-4 deficiency exacerbates brain injury and neurological deficits in acute timeframes after transient middle cerebral artery occlusion (MCAO) [20], implicating IL-4 as an endogenous protective mechanism in the early stages after stroke. We also reported that IL-4 deficiency significantly impairs long-term sensorimotor and cognitive performance after ischemic injury, whereas IL-4 supplementation improves functional outcomes in two models of stroke [16]. Although IL-4 deficiency increased the loss of NeuN^+^ brain tissue at acute phases of stroke, it did not influence neuronal tissue loss at late stages [16]. These results suggest that the long-term beneficial role of IL-4 on neurological functions after stroke might be attributed to its protection of nonneuronal cells, such as white matter components. However, the potential effects of IL-4 on white matter integrity after ischemic stroke remain elusive.

In the current study, we discovered that IL-4 is essential for white matter repair after stroke. Intranasal delivery of IL-4 nanoparticles after stroke robustly promoted oligodendrogenesis, enhanced white matter integrity, and improved long-term functional recovery. Complementary studies in cell cultures and animals revealed that IL-4-mediated oligodendrogenesis was achievable even in the absence of microglia/macrophages in vitro or after microglia/macrophage significant depletion in vivo. Furthermore, IL-4 directly promoted OPC differentiation into mature oligodendrocytes in a peroxisome proliferator-activated receptor gamma (PPARγ)-dependent manner. These collective findings suggest that activation of the IL-4/PPARγ signaling cascade may represent a novel recovery-enhancing strategy to promote long-term outcomes after ischemic stroke.

## Results

### IL-4 deficiency impairs white matter integrity after ischemic brain injury

**Fig 1:**
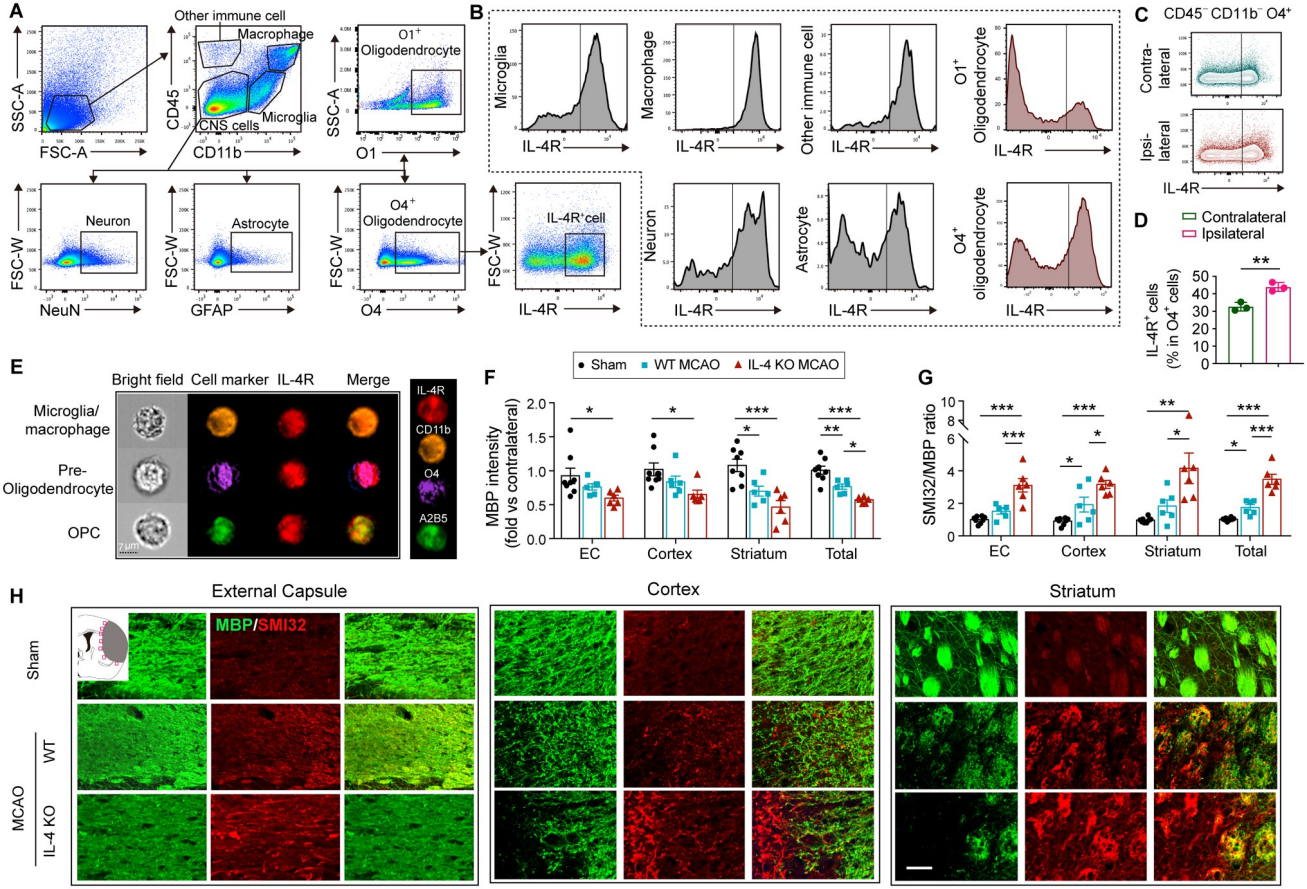
IL-4 improves white matter integrity upon demyelination. Transient focal ischemia was induced in WT and IL-4 KO mice by 60-min MCAO. (A-D) Flow cytometric analysis of IL-4R in brain cells of WT mice 3 d after MCAO. (A) Representative gating strategy for microglia (CD45^intermediate^CD11b^+^), macrophages (CD45^high^CD11b^+^), astrocytes (CD45^−^GFAP^+^), preoligodendrocytes (CD45^−^O4^+^), oligodendrocytes (CD45^−^O1^+^), neurons (CD45^−^NeuN^+^), and other immune cells (CD45^+^CD11b^−^). (B) MFI of IL-4Rα. (C-D) The expression of IL-4Rα on O4^+^ oligodendrocytes. *n* = 3 mice. *p < 0.05. Student’s *t* test. (E) ImageStream analysis demonstrated IL-4Rα expression on CD11b^+^ microglia/macrophages, O4^+^ preoligodendrocytes, and A_2_B_5_^+^ OPCs. (F-H) Double immunostaining for MBP and SMI32 14 d after MCAO or sham operation. The fluorescence intensity of MBP (F) and the ratio of SMI32 to MBP immunofluorescence intensity (G) in ipsilesional EC, cortex, striatum, and the total for all three areas quantified. *n* = 6–8/group. Data are normalized to the intensities of contralateral hemispheres. *p < 0.05, **p < 0.01, ***p < 0.001. One-way ANOVA and Bonferroni post hoc. (H) Representative images for MBP (green) and SMI32 (red) double immunostaining in the peri-infarct areas in brains from sham, WT MCAO mice, and IL-4 KO MCAO mice 14 d after MCAO. Scale bar: 50 μm. Data associated with this figure can be found in the supplemental data file (S1 Data). CNS, central nervous system; EC, external capsule; FSC-W, forward scatter width; GFAP, glial fibrillary acidic protein; IL-4R, interleukin-4 receptor α; KO, knockout; MBP, myelin basic protein; MCAO, middle cerebral artery occlusion; MFI, mean fluorescence intensity; OPC, oligodendrocyte progenitor cell; SSC-A, side scatter area; WT, wild-type.

To explore the potential cellular targets for IL-4, we used flow cytometry to assess expression of IL-4Rα on various types of cells in the ischemic brain. Consistent with previous reports [15, 21], IL-4Rα was expressed on CD45^−^CD11b^−^GFAP^+^ astrocytes, CD45^−^CD11b^−^NeuN^+^ neurons, CD45^intermediate^CD11b^+^ microglia, CD45^high^CD11b^+^ macrophages, and other infiltrated immune cells (CD45^+^CD11b^−^) 3 d after 60-min MCAO (Fig 1A and 1B). Notably, IL-4Rα was also expressed in oligodendrocyte lineage cells (Fig 1A–1D). Different markers, including A_2_B_5_, O4, and O1/galactocerebroside (GalC), were used to identify oligodendrocytes at different differentiation stages. IL-4Rα was detected in CD45^−^CD11b^−^O4^+^ cells (Fig 1A–1D), which were identified as preoligodendrocytes and premyelinating oligodendrocytes, and CD45^−^CD11b^−^O1^+^ cells (Fig 1A and 1B), which were identified as premyelinating oligodendrocytes or mature oligodendrocytes [22]. The percentages of IL-4Rα^+^O4^+^ cells were higher in the ipsilateral ischemic hemisphere (Fig 1C and 1D), suggesting a potential direct effect of IL-4 on white matter cells. ImageStream analyses confirmed that IL-4Rα was expressed on both A_2_B_5_^+^ OPCs and O4^+^ preoligodendrocytes/premyelinating oligodendrocytes, as well as CD11b^+^ microglia/macrophages in the ischemic hemisphere (Fig 1E and S1A–S1C Fig).

In light of these results, we evaluated whether IL-4 influenced white matter integrity after ischemic stroke. WT and IL-4 KO mice were subjected to transient focal ischemia induced by 60-min MCAO. Dual staining for myelin basic protein (MBP, a major myelin protein) and SMI32 (a marker of demyelinated axons) was performed to assess the lesion in white matter (Fig 1F–1H). The overall immunofluorescence intensity of MBP staining decreased in the infarct border along three white matter–rich brain regions (the external capsule [EC], the cortex, and the striatum) (see “total” values in panel of the right of Fig 1F) at 14 d after MCAO, indicating a significant loss of myelin protein after stroke. Additionally, the overall immunofluorescence of SMI32 increased in these three regions, resulting in a significant increase in the SMI32/MBP ratio in WT mice after stroke (Fig 1G). IL-4-deficient mice displayed exacerbation of the loss in MBP (Fig 1F) and a further increase in the SMI32/MBP ratio compared to WT mice (Fig 1G), suggesting that IL-4 is important in maintaining axonal myelination or promoting axonal remyelination after stroke.

**Fig 2:**
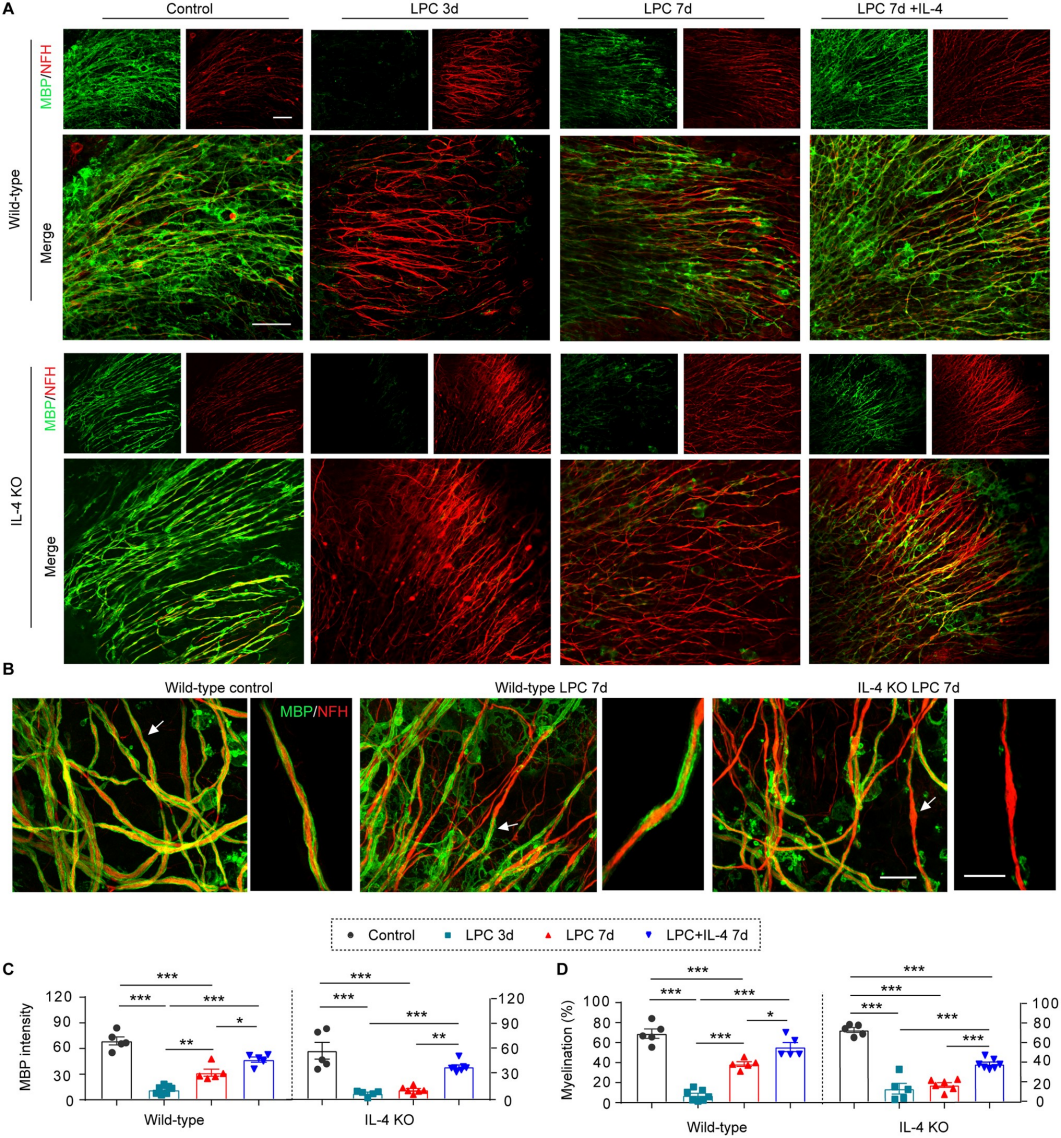
IL-4 promotes remyelination after LPC-induced demyelination in organotypic cultures. LPC (500 μg/mL) was added in WT or IL-4 KO organotypic cultures for 17 h to induce demyelination. IL-4 (20 ng/mL) or vehicle was added after LPC removal. Culture slices were fixed 3 d and 7 d after LPC treatment. Control cultures were maintained in normal culture media for 7 d. (A) MBP (green) and NFH (red) staining was performed to label myelin and axonal fibers, respectively. Scale bar: 50 μm. (B) MBP and NFH double staining and Imaris 3D reconstruction. Arrows indicate myelination of individual axonal fibers that are magnified in the images to the right. Scale bar: 15 μm. (C) Myelin integrity was measured as the intensity of MBP immunostaining. (D) The myelination index was calculated as the % MBP^+^ myelin coverage along the entire NFH^+^ fibers. *n* = 5–7 slices/group. *p < 0.05, **p < 0.01, ***p < 0.001. One-way ANOVA and Bonferroni post hoc. Data associated with this figure can be found in the supplemental data file (S1 Data). IL-4, interleukin-4; KO, knockout; LPC, lysophosphatidylcholine/lysolecithin; MBP, myelin basic protein; NFH, neurofilament heavy; WT, wild-type.

Stroke impacts both gray and white matter. In order to confirm the effect of IL-4 in white matter, we explored the remyelination capacity of IL-4 in a model of lysophosphatidylcholine/lysolecithin (LPC)-mediated demyelination using ex vivo organotypic cerebellar slice cultures. This toxicant-based experimental model of demyelination, although not attempting to fully mimic a disease with complex etiology and pathogenesis, has proven extremely useful for understanding the biology of remyelination [23]. Slices collected from WT and IL-4 KO mice exhibited comparable extents of demyelination, as revealed by the loss of MBP staining along neurofilament-heavy (NFH^+^) axonal fibers, at 3 d after LPC (Fig 2A). Partial remyelination was achieved by 7 d following LPC in WT brain slices, but IL-4 deficiency significantly delayed remyelination (Fig 2A and 2B), as shown by lower MBP staining intensity (Fig 2C) and less MBP^+^ axonal coverage (Fig 2D). IL-4 supplementation significantly enhanced remyelination in WT brain slices and rescued remyelination capacities in IL-4 KO brain slices (Fig 2A, 2C and 2D).

### Intranasal delivery of IL-4 nanoparticles promotes white matter integrity after stroke

**Fig 3:**
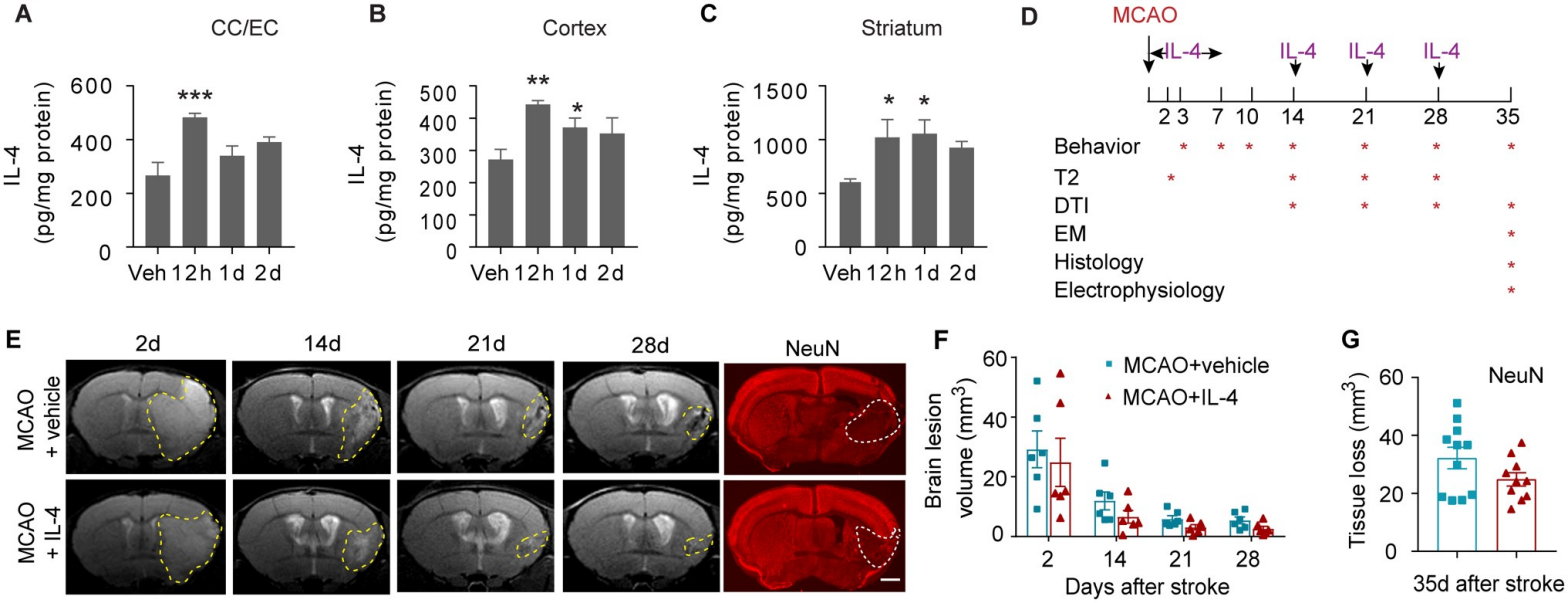
Intranasal delivery of IL-4 nanoparticles increases IL-4 levels in the brain but shows minimal effect on neuronal tissue loss. IL-4 nanoparticles (50 μg/kg body weight) or an equivalent volume of vehicle (“Veh”) were applied intranasally. Brain tissues were collected at 12 h, 1 d, and 2 d after IL-4 delivery. Brain IL-4 levels were measured in the CC and EC (A), cortex (B), and striatum (C) using ELISA. *n* = 3–4/group. *p < 0.05, **p < 0.01, ***p < 0.001, versus vehicle-treated mice. One-way ANOVA and post hoc LSD tests. (D) Experimental design for Figs 3–7. (E) Longitudinal T_2_-weighted images demonstrate axial views of the same live animal brains at 2 d, 14 d, 21 d, and 28 d after MCAO. NeuN (red) immunostaining was performed 35 d after stroke. The corresponding planes of the same animals are displayed. Scale bar for NeuN images: 1 mm. (F) Brain lesion volume was assessed on T_2_-weighted images. *n* = 6/group. Two-way ANOVA followed by Bonferroni post hoc. (G) Tissue loss was measured on NeuN-stained brain slices. *n* = 10–11/group. Student’s *t* test. Data associated with this figure can be found in the supplemental data file (S1 Data). CC, corpus callosum; DTI, diffusion tensor imaging; EC, external capsule; EM, electron microscopy; IL-4, interleukin-4; LSD, least significant difference; MCAO, middle cerebral artery occlusion.

IL-4 liposome nanoparticles were prepared to facilitate the penetration of IL-4 into the ischemic brain. The mean particle size of IL-4 protein-loaded liposome was 122.6 ± 2.4 nm with a polydispersity index of approximately 0.212 ± 0.004 and a zeta potential of 4.05 ± 1.35 mV, confirming high stability of the nanodispersion. The IL-4 nanoparticles were applied through the nasal route (50 μg/kg body weight), which has been increasingly utilized for CNS drug delivery because of its capacity to bypass the blood-brain barrier and avoid first-pass metabolism [24]. Intranasal administration of IL-4 nanoparticles effectively increased IL-4 protein levels in the corpus callosum (CC) and EC, cerebral cortex, and striatum 12 h after IL-4 application (Fig 3A–3C). IL-4 remained elevated in cortex and striatum until 24 h after delivery (Fig 3B and 3C).

C57BL/6J mice were subjected to 60-min MCAO and randomly assigned to receive IL-4 nanoparticle (50 μg/kg body weight) or control nanoparticle (vehicle) treatment. Nanomaterials were intranasally delivered at 6 h after MCAO and repeated at 1–7 d, 14 d, 21 d, and 28 d after MCAO (Fig 3D). Magnetic resonance imaging (MRI) on these animals was performed serially at 9.4 T on days 2, 14, 21, and 28 after MCAO. T_2_-weighted images showed no significant difference in the brain lesion volume of IL-4- versus vehicle-treated mice (Fig 3E and 3F), indicating comparable gross tissue damage across treatment groups. Histological staining of the neuronal marker NeuN further demonstrated comparable tissue loss 35 d after stroke in IL-4- and vehicle-treated mice (Fig 3E and 3G).

**Fig 4:**
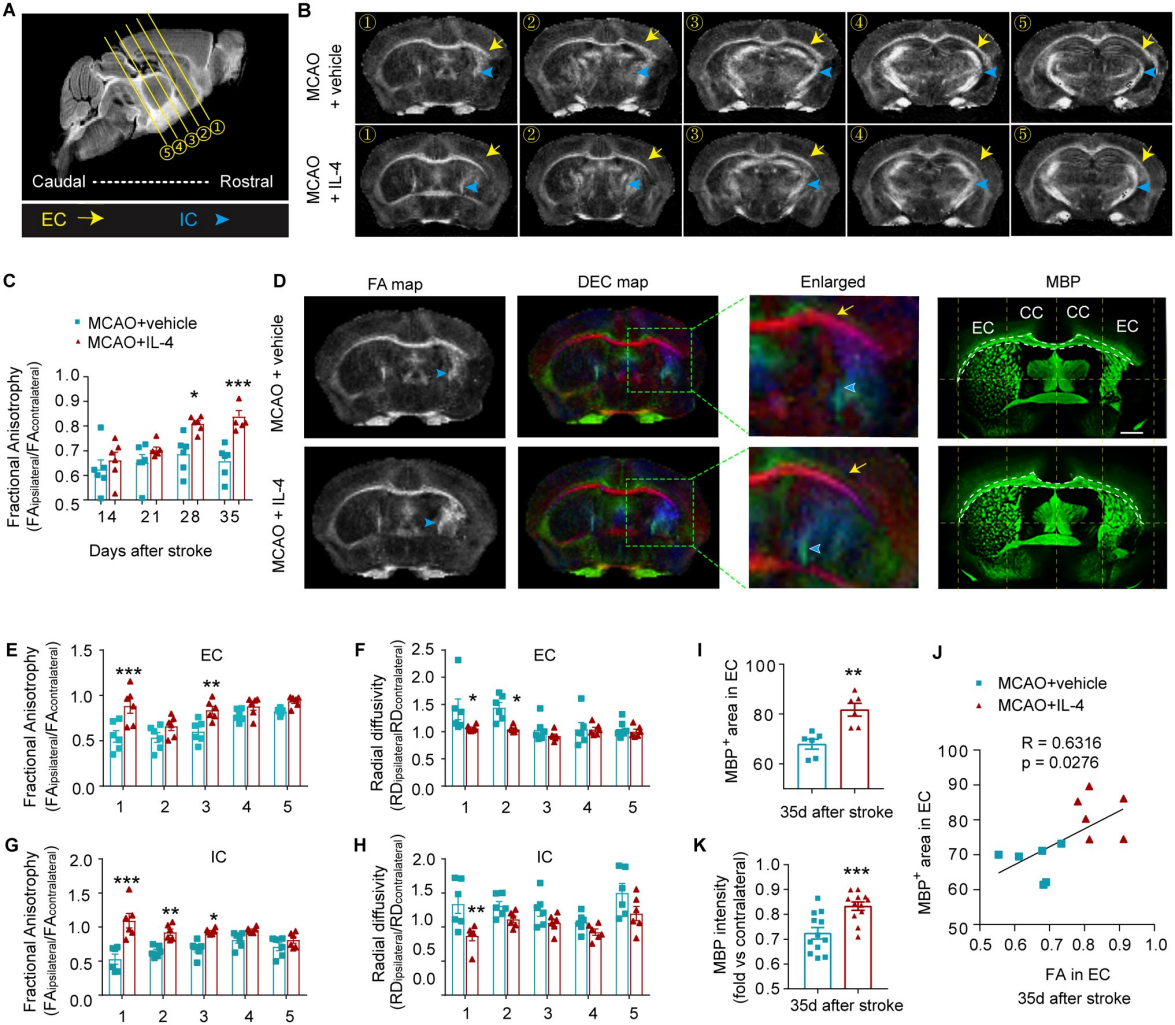
IL-4 treatment after stroke enhances white matter integrity. (A) A sagittal section showing the locations of 5 representative scanning planes from rostral to caudal. (B) Representative axial views (5 sections from rostral to caudal) of FA map 35 d after stroke. Yellow arrows indicate the EC. Blue arrow heads indicate the IC. (C) Quantification of FA values in the EC at different days after MCAO. Data are expressed as the ratio of FA values in the ipsilateral (lesioned) side to the FA values in the nonlesioned contralateral hemispheres. *n* = 6/group. *p < 0.05, ***p < 0.001 versus vehicle at indicated time points. Two-way repeated-measures ANOVA and Bonferroni post hoc. (D) Representative FA map and DEC map of the same ex vivo brains 35 d after stroke. MBP (green) immunostaining was performed on the same brains. Scale bar: 1 mm. (E-H) Quantification of FA and RD in the EC (E and F) and IC (G and H) at five axial levels from rostral to caudal. *n* = 6/group. *p < 0.05, **p < 0.01, ***p < 0.001 versus MCAO + vehicle at the same plane of section. Two-way ANOVA and Bonferroni post hoc. (I) The areas of the EC were assessed on MBP-stained brain sections. *n* = 6/group. (J) The Pearson correlation between the FA value and histological analysis of myelin in the EC. *n* = 6/group. (K) Quantification of MBP intensity in peri-infarct areas. Data are calculated as fold change compared to corresponding contralateral areas. *n* = 12/group. **p < 0.01, ***p < 0.001 versus MCAO + vehicle, Student’s *t* test. Data associated with this figure can be found in the supplemental data file (S1 Data). CC, corpus callosum; DEC, directionally encoded color; EC, external capsule; FA, fractional anisotropy; IC, internal capsule; IL-4, interleukin-4; MBP, myelin basic protein; MCAO, middle cerebral artery occlusion; RD, radial diffusivity.

Serial whole-brain in vivo diffusion tensor imaging (DTI) scans were collected 14 d, 21 d, and 28 d after MCAO. The same animals were then killed at 35 d after stroke, and the fixed brains were subjected to ex vivo DTI scanning (Fig 4A and 4B, S2A and S2B Fig). Longitudinal observation of fractional anisotropy (FA) maps and calculation of the ratio of FA(ipsilateral)/FA(contralateral) demonstrated progressive white matter injury (ratio < 1) with a slow recovery in the EC of vehicle-treated stroke mice at 14–35 d after stroke, which was significantly improved by day 28 following stroke in IL-4-treated mice (Fig 4C). This trend was observed until day 35 in the ex vivo FA values (Fig 4C). Further analysis of the 35 d DTI images revealed that IL-4 treatment enhanced white matter integrity, as revealed by elevated FA values in the white matter–enriched EC and internal capsule (IC) (Fig 4D, 4E and 4G). In addition, IL-4 treatment significantly reduced radial diffusivity (RD, λ_⊥_) in the EC (Fig 4F) and IC (Fig 4H), which likely indicates improved myelin integrity [25]. No significant differences between IL-4 and vehicle treatment groups were observed in axial diffusivity (AD, λ_||_), a measurement for axonal integrity (S2C and S2D Fig).

To further explore the biological basis of these DTI results, we compared the DTI results with immunohistochemical staining of MBP performed in the same animals. There were remarkable similarities between the FA maps and MBP images (Fig 4D, 4I and 4J). IL-4 treatment significantly increased the MBP^+^ area in the EC (Fig 4D and 4I). There was a significant positive correlation between the MBP^+^ areas and the FA values in the EC (Fig 4J). The quantification of MBP staining confirmed a significant increase in MBP intensity in the ischemic brains of IL-4-treated mice compared to vehicle-treated mice (Fig 4K).

### IL-4 post-treatment improves the structural and functional integrity of myelinated fibers after stroke

**Fig 5:**
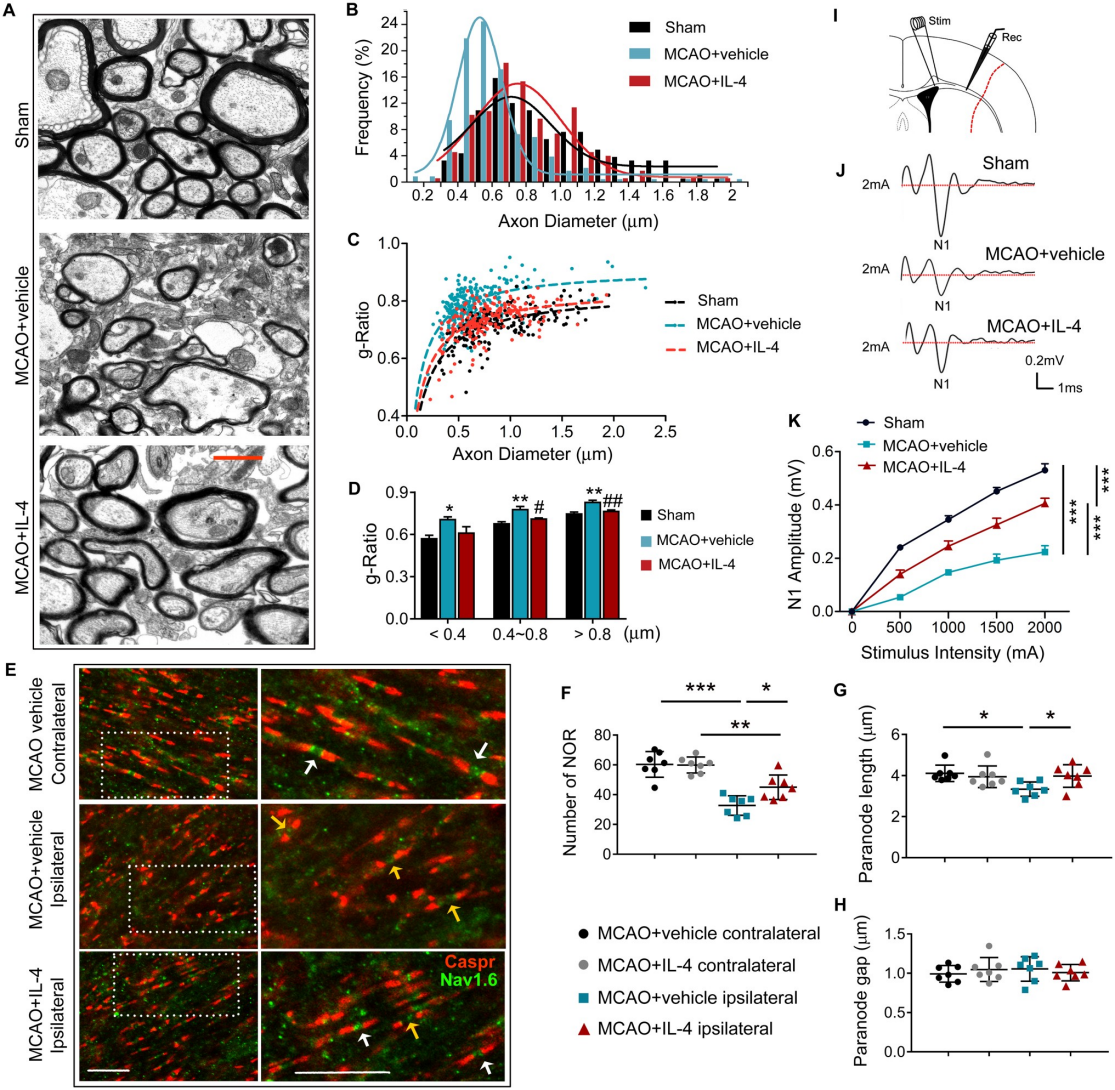
IL-4 post-treatment improves the anatomical integrity and physiological function of myelinated fibers after stroke. (A-D) TEM ultrastructural analyses of myelin integrity in the EC of sham, MCAO+vehicle, and MCAO+IL-4 groups at 35 d after surgery. (A) Representative TEM images show thinner myelin sheaths at a demyelination site in the EC after stroke. IL-4 treatment increased myelin thickness. Scale bar: 1 μm. (B) Frequency histograms of axon diameter. (C) Scatter plots of *g*-ratio as a function of axon diameter in sham mice or stroke mice treated with vehicle or IL-4. (D) The *g*-ratio of myelinated axons with respect to axon diameter at 0.4-μm intervals for sham mice or stroke mice treated with vehicle or IL-4. *n* = 3/group. *p < 0.05, **p < 0.01 versus sham; #*p* < 0.05, ##*p* < 0.01 versus MCAO + vehicle. One-way ANOVA followed by Bonferroni post hoc. (E-H) Caspr and NaV1.6 immunostaining in the EC of MCAO+vehicle and MCAO+IL-4 groups at 35 d after surgery. (E) Representative images of Caspr (red) and NaV1.6 (green) immunostaining. Scale bar: 10 μm. (F) Quantification of numbers of NORs. (G) Quantification of the paranode (Caspr^+^) length. (H) The length of the paranode gap was quantified as the length between a pair of Caspr^+^ sites. *N* = 7/group. *p < 0.05, **p < 0.01, ***p < 0.001. One-way ANOVA followed by Bonferroni post hoc. (I-K) Electrophysiological analyses of brain slices prepared from sham, MCAO+vehicle, and MCAO+IL-4 mice 35 d after MCAO. (I) Schematic illustration of the position of the stimulating (“Stim”) and recording (“Rec”) electrodes for CAP measurements in CC/EC. (J) Representative curves of CAPs in myelinated N1 fibers. (K) Quantification of amplitude of evoked CAPs in myelinated N1 fibers. *N* = 6/group. ***p < 0.001. Two-way ANOVA repeated measures followed by Bonferroni post hoc. Data associated with this figure can be found in the supplemental data file (S1 Data). CAP, compound action potential; Caspr, contactin-associated protein; CC, corpus callosum; EC, external capsule: IL-4, interleukin-4; MCAO, middle cerebral artery occlusion; NOR, nodes of Ranvier; TEM, transmission electron microscopy.

To determine whether IL-4 can improve axonal myelination after stroke, myelin thickness was measured in the EC using transmission electron microscopy (TEM) (Fig 5A). Frequency histograms revealed much greater reduction of axon diameters in the MCAO + vehicle group compared to the MCAO + IL-4 and sham groups, suggesting higher axonal degeneration in stroke mice without IL-4 treatment (Fig 5B). Scatter plots of the *g*-ratio (the ratio of the inner axonal diameter to the total outer diameter of myelinated fiber) as a function of axon diameter in sham mice or stroke mice treated with vehicle or IL-4 revealed significant differences in myelin thickness (average *g*-ratio: sham = 0.72 [*n* = 183 axons from 3 animals]; MCAO + vehicle = 0.79 [*n* = 233 axons from 3 animals]; MCAO + IL-4 = 0.73 [*n* = 176 axons from 3 animals]) (Fig 5C). Significant increases in the *g*-ratio were observed in both low- and high-caliber axons at 35 d after MCAO, indicating a reduction in myelin thickness (Fig 5D). IL-4 post-treatment significantly reduced the *g*-ratio of the middle-sized (diameter = 400–800 nm) and large (diameter > 800 nm) axonal fibers, suggesting that IL-4 may maintain axonal myelination or promote axonal remyelination after stroke. The *g*-ratio in small axonal fibers (diameter < 400 nm) displayed a trend toward reduction with IL-4 treatment (Fig 5D).

Saltatory conduction of action potentials along myelinated axons relies on normal organization of the nodes of Ranvier (NOR), which are rich in sodium and potassium ion channels. Double immunofluorescent analyses of Nav1.6 (a nodal sodium channel protein) and contactin-associated protein (Caspr, a paranodal cell adhesion protein) revealed the NOR in the EC (Fig 5E). Typical NOR are characterized by punctate Nav1.6 staining at nodes between the paranodal staining of Caspr. The total numbers of normal NOR decreased significantly after stroke in the ipsilateral EC compared to the contralateral EC (Fig 5F), and this was accompanied by a decrease in the Caspr^+^ paranode length (Fig 5G). IL-4 treatment significantly increased the number of NOR and the paranode length in the ischemic EC (Fig 5F and 5G), suggesting an improvement in NOR structure. The paranodal gap between pairs of Caspr staining remained unchanged between groups (Fig 5H).

As IL-4 enhanced the structural integrity of white matter after MCAO, we further evaluated white matter function by measuring the transmission of action potentials in the CC and EC (Fig 5I). Evoked compound action potentials (CAPs) display an early peak representing fast-conducting myelinated axons (N1, Fig 5J) [26]. The amplitude of the N1 segment was significantly reduced 35 d after MCAO (Fig 5K), indicating impaired conduction through myelinated axons. IL-4-treated mice exhibited less reduction in the N1 amplitude after MCAO, suggesting myelin preservation or improved remyelination of axons compared to the vehicle-treated subjects.

### IL-4-afforded white matter protection is associated with superior long-term functional recovery after stroke

**Fig 6:**
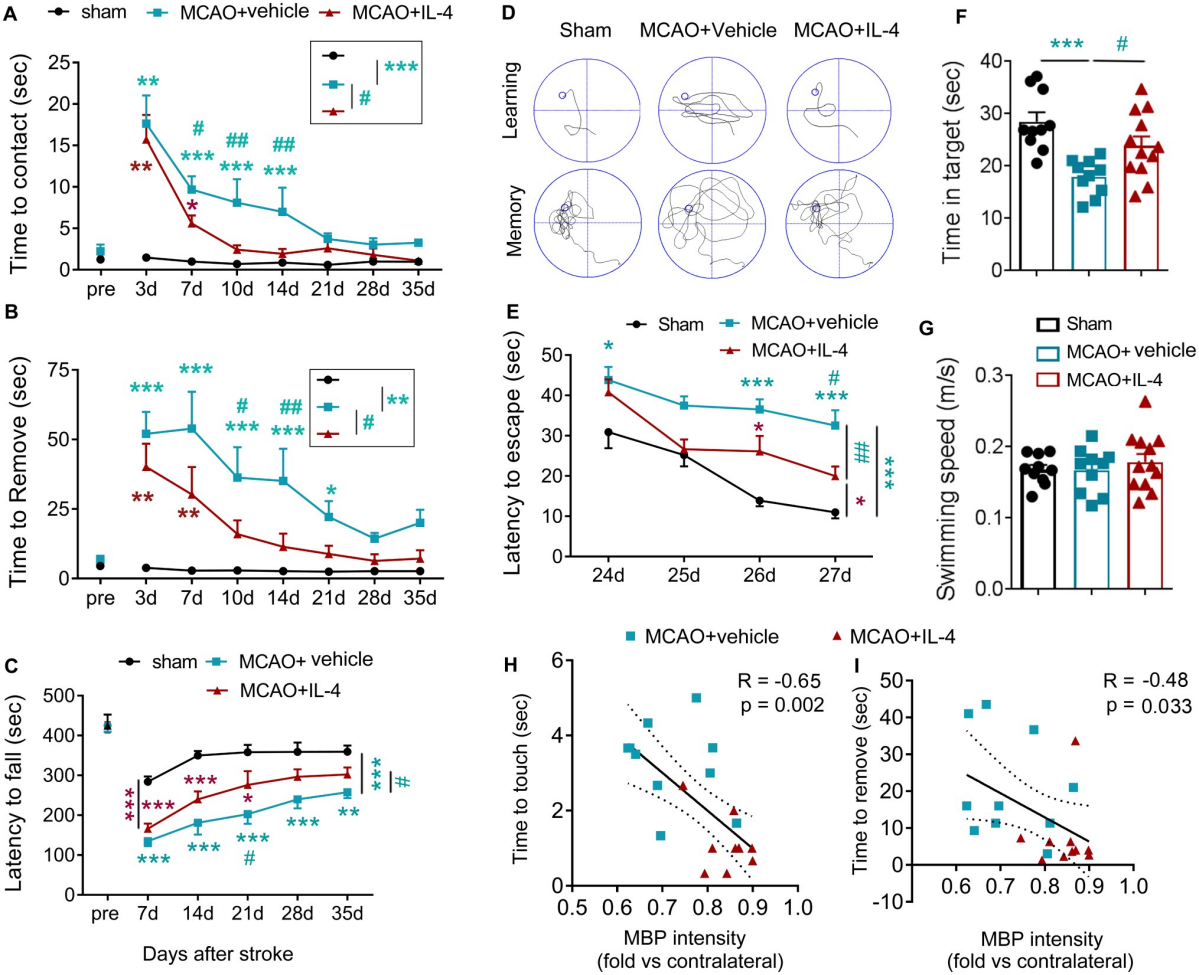
Poststroke administration of IL-4 improves long-term neurological functions after MCAO. Mice received intranasal infusions of IL-4 (50 μg/kg) or vehicle 6 h after 60-min MCAO and at daily intervals on days 1–7 and then every 7 d until 28 d after stroke. (A-C) Sensorimotor functions were assessed up to 35 d after MCAO or sham operation. (A-B) Adhesive removal test. The latencies to contact (A) and remove (B) the tapes were recorded. *n* = 10–14/group. (C) Rotarod test. The latency to fall or spin around on the rungs was quantified. *n* = 7–9/group. (D-G) Long-term cognitive functions were evaluated in the Morris water maze. Panel D shows representative images of the swim paths of mice in each group while the platform was present (learning phase) or removed (memory phase). IL-4-treated mice showed improved cognitive functions, as reflected by shorter escape times (E, learning), and extension of the time spent in the target quadrant (F, memory) with no change in swim speed (G). *n* = 10–12/group. *p < 0.05, **p < 0.01, ***p < 0.001, versus sham or as indicated. #*p* < 0.05, ##*p* < 0.01, ###*p* < 0.001 MCAO+IL-4 versus MCAO+vehicle or as indicated. Two-way ANOVA repeated measurement or one-way ANOVA and Bonferroni post hoc. (H-I) Spearman correlation between MBP intensity at 35 d after MCAO and average latency to contact or remove adhesive tape from the lesioned forelimb at 35 d after MCAO. Data associated with this figure can be found in the supplemental data file (S1 Data). IL-4, interleukin-4; MBP, myelin basic protein; MCAO, middle cerebral artery occlusion.

Sensorimotor and cognitive functions were assessed by investigators blinded to group assignments. As shown in Fig 6A and 6B, IL-4-treated and vehicle-treated stroke mice showed comparable reductions in latencies to contact and remove adhesive tape from the paw at 3 d after stroke, suggesting similar initial deficits in dexterity across groups. However, at later time points starting from 7–10 d after stroke, IL-4 treatment reduced the time spent touching and removing tapes in stroke mice, culminating in improved performance in the adhesive removal test (Fig 6A and 6B). Long-term sensorimotor performance in the rotarod test after MCAO was also improved in IL-4-treated mice (Fig 6C). Furthermore, IL-4-treated mice exhibited long-term improvements in cognitive function in the Morris water maze test, as manifested by a reduction in the latency to find the hidden platform (improved spatial learning, Fig 6D and 6E) and increased time spent in the target quadrant when the platform was removed (improved memory, Fig 6D and 6F). There was no difference in swimming speed among different treatment groups, indicating similar gross motor skills across groups (Fig 6G).

Next, we measured the potential correlation between white mater integrity and behavioral performance after stroke in vehicle or IL-4-treated mice. The results revealed significant negative correlations between MBP intensity and the latency to touch (Fig 6H) and remove (Fig 6I) the adhesive tape at 35 d after stroke, confirming an association between histological observations of white matter integrity and long-term functional recovery of behavioral endpoints.

### IL-4 treatment promotes oligodendrogenesis partially independent of microglia/macrophages

**Fig 7:**
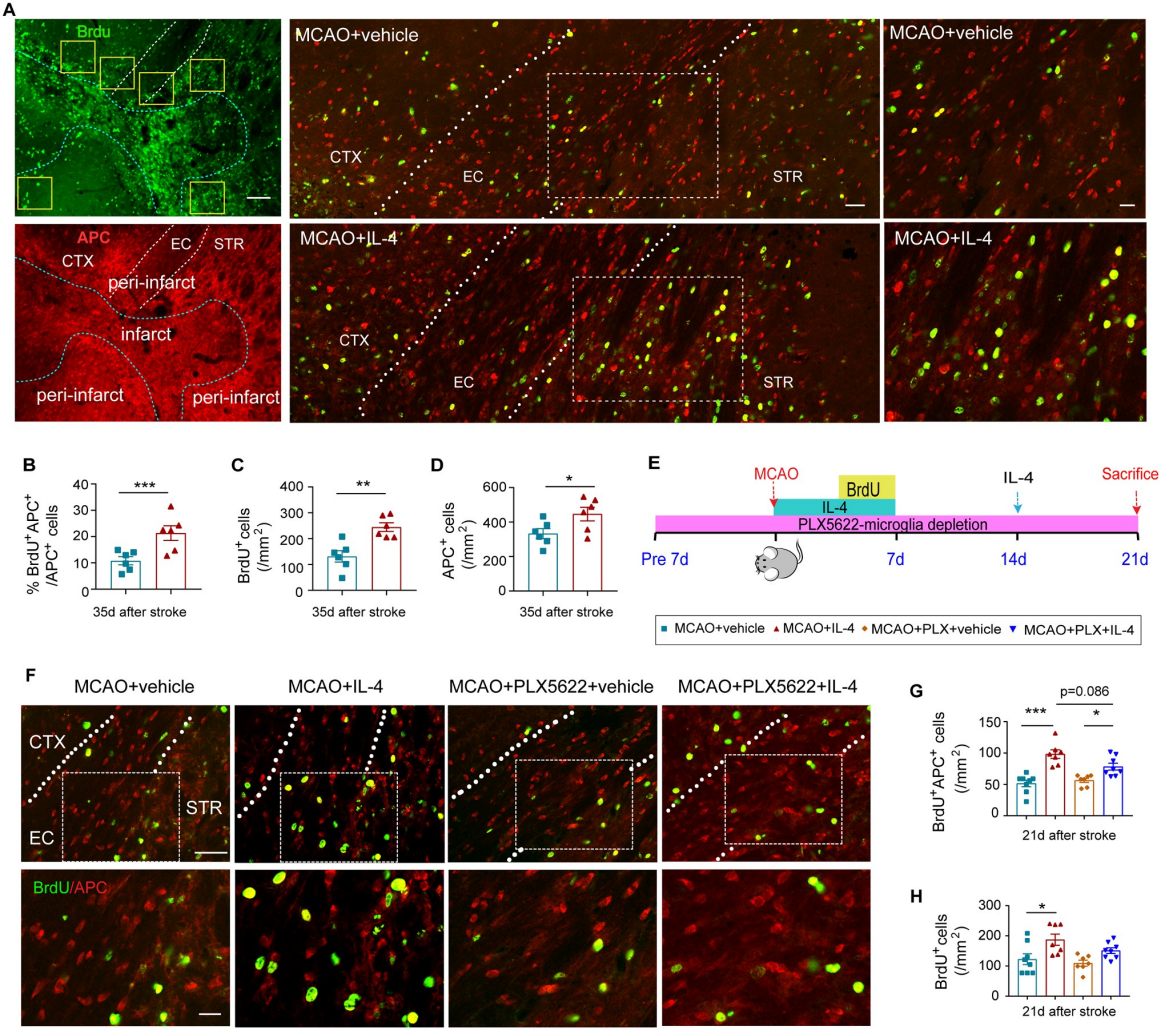
IL-4 treatment promotes oligodendrogenesis in a microglia/macrophage-independent manner. (A-D) Mice received intranasal infusions of IL-4 nanoparticles (50 μg/kg) or vehicle 6 h after MCAO and at daily intervals on days 1–7 and then every 7 d until 28 d after stroke. (A) Representative images of BrdU (green) and APC (red) immunostaining 35 d after cerebral ischemia. Scale bars:100 μm (left), 50 μm (middle), and 25 μm (right). The dotted blue lines delineate the border of infarct lesion. The dotted white lines trace the boundaries of the EC. Yellow squares indicate the areas of imaging in the peri-infarct area. (B) Quantification of the ratio of BrdU^+^APC^+^ cells among total APC^+^ cells in the ischemic areas, corresponding to images in panel A. (C) Quantification of the number of BrdU^+^ cells in the peri-infarct areas. (D) Quantification of the number of APC^+^ cells in the peri-infarct areas. *n* = 6/group. *p < 0.05, **p < 0.01, ***p < 0.001. Student’s *t* test. (E-H) Microglia/macrophages were depleted by dietary intake of PLX5622 7 d before 60-min MCAO. Mice received intranasal infusions of IL-4 (50 μg/kg) or vehicle 6 h after MCAO and at daily intervals on days 1–7 and then every 7 d until 14 d after stroke. (E) Experimental design. (F) Representative images of BrdU (green) and APC (red) immunostaining 21 d after cerebral ischemia. The dotted white lines trace the boundaries of the EC. White squares indicate the areas enlarged in the images below. Scale bars: 50 μm (upper) and 10 μm (lower). (G) Quantification of the number of BrdU^+^APC^+^ cells in the peri-infarct areas. (H) Quantification of the number of BrdU^+^ cells in the peri-infarct areas. *n* = 7–8/group. *p < 0.05, ***p < 0.001. One-way ANOVA followed by Bonferroni post hoc. Data associated with this figure can be found in the supplemental data file (S1 Data). APC, adenomatous polyposis coli; BrdU, 5-bromo-2′-deoxyuridine; CTX, cortex; EC, external capsule; IL-4, interleukin-4; MCAO, middle cerebral artery occlusion; STR, striatum.

Successful differentiation of OPCs into myelinating oligodendrocytes is important for remyelination [11]. Thus, we compared the regeneration of myelin-producing oligodendrocytes in MCAO mice with or without IL-4 treatment. 5-bromo-2′-deoxyuridine (BrdU) was injected at 3–6 d after MCAO to label newly generated cells. Mature oligodendrocytes were recognized by immunostaining with anti-adenomatous polyposis coli (APC) (or CC1) antibodies [27]. Compared to vehicle-treated mice, a significantly higher ratio of BrdU^+^APC^+^ newly generated oligodendrocytes was observed in IL-4-treated mice 35 d after MCAO (Fig 7A and 7B). The total numbers of BrdU^+^ cells and the total numbers of APC^+^ cells were also significantly increased in IL-4-treated mice compared to vehicle-treated mice (Fig 7C and 7D). It has been reported that higher concentrations of the APC antibody may detect some populations of astrocytes in certain pathological conditions [27]. However, no APC signal was detected in glial fibrillary acidic protein (GFAP)^+^ astrocytes in the ischemic brain in our preclinical model of stroke (S3 Fig).

IL-4 is known to promote microglia M2 polarization, which in turn enhances OPC differentiation and remyelination [28]. We observed in our model that IL-4 treatment robustly promoted microglia/macrophage polarization toward an anti-inflammatory phenotype (CD206^+^Iba1^+^) at 7 and 14 d after stroke (S4 Fig). Microglia depletion through genetic targeting or pharmacological treatment is a widely used approach to evaluate the contribution of this population to various biological processes [29]. Therefore, we chose PLX5622, an inhibitor of colony-stimulating factor 1 receptor, to deplete microglia/macrophages and identify their role in IL-4-afforded oligodendrogenesis (Fig 7E) [30]. Consistent with previous reports [30], PLX5622 achieved approximately 90% depletion efficacy within a short period. Dietary intake of PLX5622 for 7 d dramatically reduced the number of microglia in the normal brain (S5A Fig). Continuation on the PLX5622 diet maintained low numbers of microglia/macrophages in the brain 21 d after sham or MCAO surgery (28 d after initiation of PLX5622 diet) (S5A–S5C and S5E Fig). The number of neurons remained statistically similar in microglia/macrophage-depleted mice and microglia/macrophage-competent mice (S5A, S5B, S5D and S5F Fig). The PLX5622 diet also selectively reduced the number of CD3^−^CD19^−^Gr1^−^CD11b^+^F4/80^+^ macrophages in the blood (S5G and S5H Fig). Significant depletion of microglia/macrophages did not change the number of BrdU^+^APC^+^ cells or total BrdU^+^ cells compared to microglia/macrophage-competent stroke mice (Fig 7F–7H). IL-4 treatment increased the numbers of BrdU^+^APC^+^ cells or total BrdU^+^ cells after stroke in mice fed with the control diet. IL-4 treatment exhibited a trend (*p* = 0.086) toward lower capacities to increase the number of BrdU^+^APC^+^ newly generated oligodendrocytes in microglia-depleted mice versus microglia-competent mice, supporting a potential role of microglia in IL-4-afforded oligodendrogenesis. Importantly, IL-4 treatment still resulted in a significant enhancement of BrdU^+^APC^+^ oligodendrocyte regeneration in microglia/macrophage-depleted stroke mice (Fig 7F and 7G). Considering the expression of IL-4R on oligodendrocyte linage cells, these results indicate that IL-4 may exert microglia/macrophage-independent effects on oligodendrocyte regeneration after demyelination.

### Intranasal IL-4 treatment exerts minimal effects on adaptive immune cell populations

Consistent with a previous study in a model of multiple sclerosis [31], we found that repeated intranasal IL-4 administration did not change T-lymphocyte subpopulations or B lymphocytes in the blood (S6A–S6C Fig), suggesting that the nasal application of IL-4 has minimal systemic effects on these peripheral immune cells. Additionally, intranasal IL-4 did not affect the composition of infiltrated lymphocytes in the brain (S6D Fig). These data do not support the involvement of adaptive immune responses in IL-4-afforded neuroprotection.

### IL-4 directly stimulates OPC differentiation in primary OPC cultures

**Fig 8:**
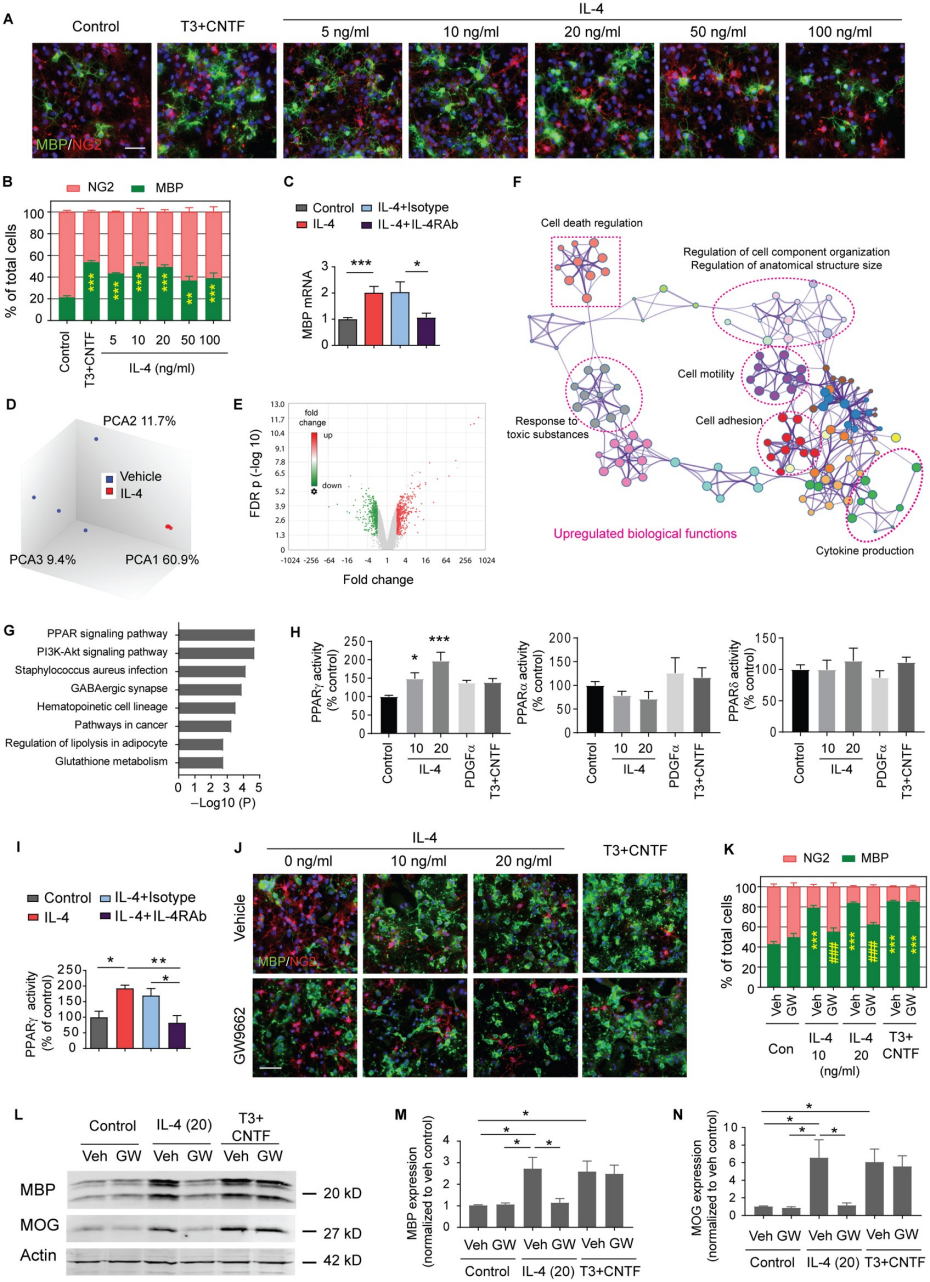
PPARγ is essential for IL-4-induced OPC differentiation in vitro. (A-C) OPCs were treated with vehicle (PBS), various concentrations of IL-4, or T3 (50 ng/mL)+CNTF (10 ng/mL) for 3 d. (A) Double staining of MBP (green) and NG2 (red). Nuclear staining with DAPI is shown in blue. Scale bar: 50 μm. (B) Quantification of MBP^+^ cells and NG2^+^ cells as percentages of total cells. Three independent experiments in duplicate. **p < 0.01, ***p < 0.001 versus control. Two-way ANOVA and Bonferroni post hoc. (C) Expression of *mbp* was assessed by qRT-PCR 3 d after vehicle or IL-4 (20 ng/mL) treatment. Pretreatment with IL-4RAbs (20 ng/mL) for 30 min blocked the IL-4-enhanced expression of *mbp* compared to isotype control antibodies (20 ng/mL). Data are fold change relative to control. *n* = 5–9/group. *p < 0.05, ***p < 0.001. Student’s *t* test. (D-G) Microarray analyses on OPCs treated with PBS or IL-4 (20 ng/mL) for 3 d. *n* = 4/group. (D) PCA of microarray expression profiles. (E) Volcano plot. (F) Network plot showing enriched GO biological processes that were up-regulated by IL-4 treatment. (G) KEGG pathways that were significantly up-regulated in IL-4-treated OPCs. (H) Quantification of PPAR DNA binding activity. *n* = 4/group. *p < 0.05, ***p < 0.001 versus control. One-way ANOVA and Bonferroni post hoc. (I) Quantification of PPARγ DNA binding activity in vehicle or IL-4 (20 ng/mL)–treated OPCs, with or without IL-4Rα neutralizing Ab or isotype IgG (20 ng/mL). *n* = 4–6/group. *p < 0.05, **p < 0.01. One-way ANOVA and Bonferroni post hoc. (J-N) OPCs were treated with the PPARγ inhibitor GW9662 (0.5 μM) or DMSO (“Veh”) for 30 min, followed by IL-4, vehicle control (PBS), or T3+CNTF for 5 d. (J) Immunostaining of MBP (green) and NG2 (red). Scale bar: 50 μm. (K) Quantification of MBP^+^ cells and NG2^+^ cells as percentages of total cells. Three independent experiments in duplicate. ***p < 0.001 versus control. ###*p* < 0.001 versus IL-4+veh. Two-way ANOVA and Bonferroni post hoc. (L) Representative western blots of MBP and MOG. (M-N) Quantification of MBP (M) and MOG (N) expression in western blots. *n* = 3/group. *p < 0.05. One-way ANOVA and Bonferroni post hoc. Data associated with this figure can be found in the supplemental data file (S1 Data). CNTF, ciliary neurotrophic factor; FDR, false discovery rate; GO, gene ontology; IgG, immunoglobulin G; IL-4, interleukin-4; IL-4RAb, IL-4 receptor α neutralizing antibody; KEGG, Kyoto Encyclopedia of Genes and Genomes; MBP, myelin basic protein; MOG, myelin oligodendrocyte glycoprotein; NG2, neuron-glia antigen 2; OPC, oligodendrocyte progenitor cell; PCA, principal component analysis; PDGFα, platelet-derived growth factor alpha; PI3K, phosphoinositide 3-kinase; PPAR, peroxisome proliferator-activated receptor; qRT-PCR, quantitative reverse transcription PCR.

Consistent with a direct effect of IL-4 on oligodendrocytes, in vitro studies showed that IL-4 at a concentration range of 5–100 ng/mL directly stimulated the differentiation of neuron-glia antigen 2 (NG2)^+^ OPCs into MBP^+^ mature oligodendrocytes without the aid of microglia (Fig 8A and 8B). IL-4 at concentrations of 10 or 20 ng/mL induced OPC differentiation that was comparable to the effects of a T3 cocktail typically used to induce OPC differentiation in vitro. The effect of IL-4 on OPC differentiation was indeed IL-4R-dependent, because specific IL-4Rα neutralizing antibodies (IL-4RAbs) blocked the IL-4-induced increase in mRNA expression of the mature oligodendrocyte marker *mbp* (Fig 8C). IL-4 at a concentration range of 5–100 ng/mL had no effect on OPC survival (S7A and S7B Fig) or proliferation (S7C Fig).

### PPARγ is essential for IL-4-induced OPC differentiation and maturation

Microarray analyses were performed to further elucidate the mechanisms underlying the promotion of oligodendrogenesis by IL-4. OPCs were treated with PBS or IL-4 (20 ng/mL) for 3 d. Total RNA extraction from cell pellets was used for the Affymetrix Clariom S rat array. Principal component analysis (PCA) showed clustering of samples in a treatment-dependent manner (Fig 8D). In total, 996 differentially expressed genes (DEGs) were identified (false discovery rate [FDR] < 0.05; and fold change > 2), out of which 495 were up-regulated and 501 were down-regulated in IL-4-treated OPCs relative to PBS-treated OPCs (Fig 8E, S1 and S2 Tables). The gene ontology (GO) enrichment analysis was then performed using the Metascape analysis [32] (http://metascape.org). The top 20 enriched GO terms in three categories (biological processes, cellular components, and molecular functions) were identified in the up-regulated DEGs by IL-4 treatment (S8A Fig). A network plot further captured the relationships between the main regulated biological processes, which were related to cell adhesion and motility, cytokine production, cell responses to external stimuli, cellular component organization, and cell death (Fig 8F). The enriched GO terms among the down-regulated DEGs in IL-4-treated OPCs represent biological processes such as developmental growth, small molecule biosynthetic process, vessel development, and responses to nutrients and peptides (S8B Fig). These results indicated changes in cell mobilization, growth, differentiation, and defense in IL-4-treated OPCs. A Kyoto encyclopedia of Genes and Genomes (KEGG) pathway analysis identified eight enriched pathways for up-regulated DEGs, of which the PPAR signaling pathway was dramatically up-regulated in IL-4-treated OPCs compared to vehicle-treated cells (Fig 8G). The PPAR transcription factor assay confirmed that PPARγ activity, but not PPARα or PPARδ activities, was up-regulated in OPCs upon IL-4 treatment (Fig 8H); these effects were abolished when IL-4Rα was blocked with a neutralizing antibody (Fig 8I). Furthermore, immunostaining (Fig 8J and 8K) and western blotting (Fig 8L–8N, and S9 Fig) demonstrated that the PPARγ inhibitor GW9662 blocked IL-4-induced expression of mature oligodendrocyte markers (MBP or myelin oligodendrocyte glycoprotein [MOG]), suggesting that PPARγ activation is essential for the OPC differentiation induced by IL-4.

**Fig 9:**
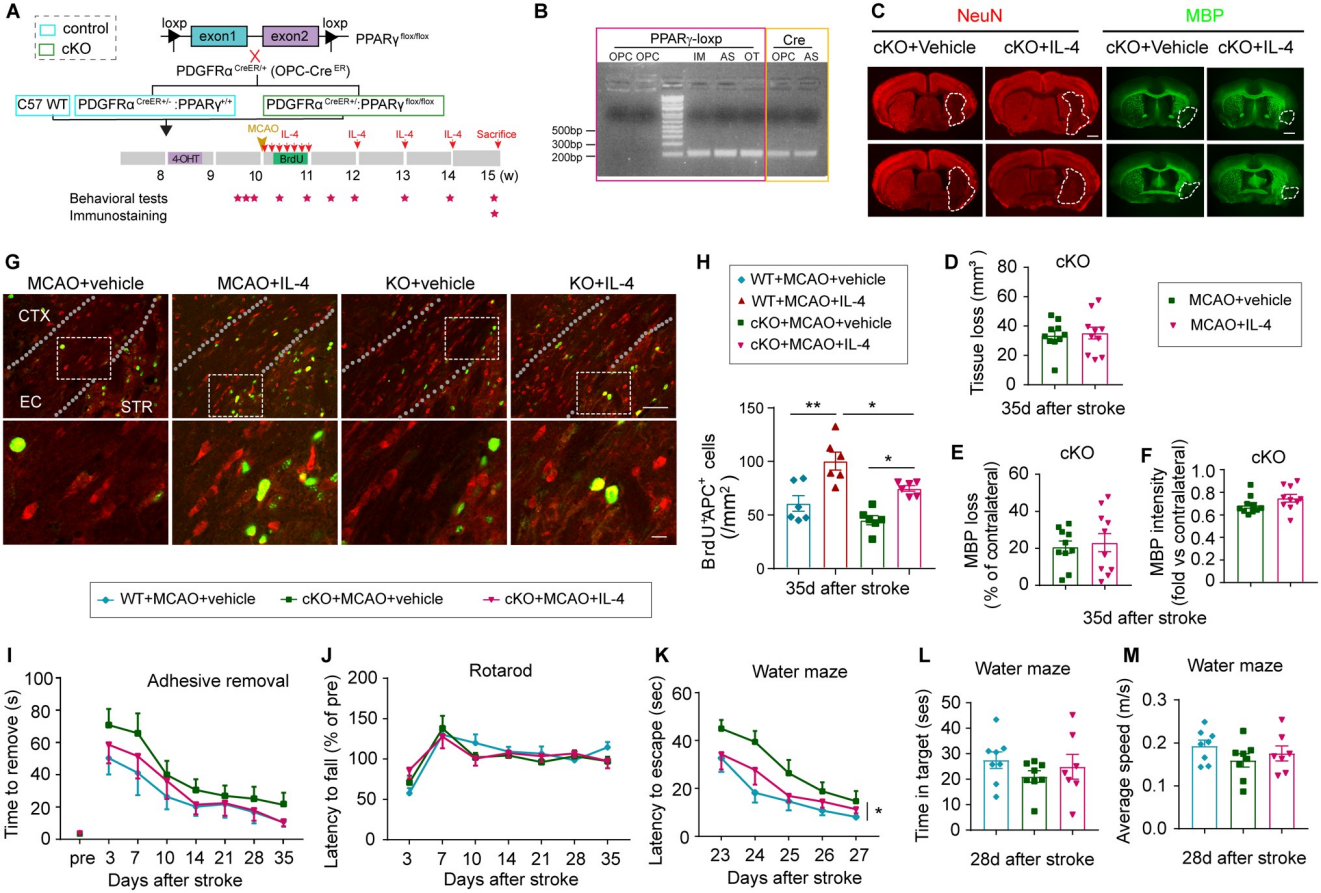
PPARγ is essential for IL-4-induced OPC differentiation and long-term functional improvements after stroke. (A) Experimental design. (B) PDGFRα^+^CD45^−^ OPC, GLAST^+^CD45^−^ AS, CD45^+^ IM, and CD45^−^GLAST^−^PDGFRα^−^ other CNS cells (“OT”) were sorted from the brains of PPARγ-OPC KO mice at 9 d after the final 4-OHT injection. The DNA extracts were subjected to PCR using primers flanking the loxp site and primers detecting the Cre cDNA. (C) Representative images of NeuN and MBP immunostaining in brain sections. Scale bar: 1 mm. (D) Neuronal tissue loss was quantified by NeuN immunostaining. *n* = 10/group. (E) White matter injury was quantified by MBP immunostaining. The data was expressed as the % loss of MBP^+^ area compared to the MBP^+^ area of contralateral hemisphere. *n* = 10/group. (F) Quantification of MBP intensity. Data are calculated as fold change compared to corresponding contralateral areas. *n* = 10/group. (G) Representative images of BrdU (green) and APC (red) immunostaining. The dotted white lines trace the boundaries of the EC. White squares indicate the areas enlarged in the images below. Scale bars: 50 μm (upper) and 10 μm (lower). (H) Quantification of the number of BrdU^+^APC^+^ cells in the ischemic areas. *n* = 6/group. *p < 0.05, **p < 0.01. One-way ANOVA and Bonferroni post hoc. (I-J) Sensorimotor functions were assessed by the adhesive removal test (I) and Rotarod test (J). *n* = 10–13/group. Two-way ANOVA repeated measurement and Bonferroni post hoc tests. (K-M) Long-term cognitive functions were evaluated in the Morris water maze. *n* = 7–8/group. *p < 0.05. Two-way repeated ANOVA (K) or one-way ANOVA (L-M) and Bonferroni post hoc. Data associated with this figure can be found in the supplemental data file (S1 Data). 4-OHT, 4-hydroxytamoxifen; APC, adenomatous polyposis coli; AS, astrocytes; BrdU, 5-bromo-2′-deoxyuridine; CNS, central nervous system; CTX, cortex; EC, external capsule; IL-4, interleukin-4; IM, immune cells; KO, knockout; MBP, myelin basic protein; MCAO, middle cerebral artery occlusion; OPC, oligodendrocyte progenitor cell; PPARγ, peroxisome proliferator-activated receptor gamma; STR, striatum; WT, wild-type.

Finally, OPC-specific PPARγ KO (PPARγ-OPC KO) mice were used to confirm the importance of PPARγ signaling in IL-4-enhanced oligodendrogenesis in an in vivo stroke model (Fig 9A). To confirm the KO of PPARγ specifically in OPCs, PDGFRα^+^CD45^−^ OPCs, GLAST^+^CD45^−^ astrocytes, CD45^+^ immune cells, and other CD45^−^GLAST^−^PDGFRα^−^ CNS cells were sorted from the brains collected from PPARγ-OPC KO mice at 9 d after 4-hydroxytamoxifen injections. The DNA extracts were subjected to PCR using primers flanking the loxp site and primers detecting the Cre cDNA. The loxp band was abolished in OPCs because of the targeted deletion after Cre-induced recombination, while remaining intact in astrocytes, immune cells, and other CNS cells sorted from the same brain (Fig 9B). The expression of Cre was detected in the OPCs of the PPARγ-OPC KO mice (Fig 9B). Deletion of PPARγ specifically in OPCs exerted no impact on IL-4R expression on O4^+^ oligodendrocytes (S10 Fig). Vehicle or IL-4 treatment in PPARγ-OPC KO mice resulted in similar neuronal tissue loss (Fig 9C and 9D) and white matter lesion (Fig 9C, 9E and 9F) 35 d after stroke. The lack of PPARγ expression in OPCs did not significantly impair oligodendrocyte replacement after stroke, as shown by comparable numbers of BrdU^+^APC^+^ cells in the ischemic brain from PPARγ-OPC KO mice and control mice at 35 d after MCAO (Fig 9G and 9H). However, the absence of PPARγ in OPCs reduced the capacity of IL-4 to increase the number of newly generated BrdU^+^APC^+^ oligodendrocytes (Fig 9G and 9H). Consistent with a reduced capacity to promote oligodendrogenesis, IL-4 failed to improve long-term sensorimotor (Fig 9I and 9J) or cognitive (Fig 9K–9M) deficits after stroke in the absence of PPARγ.

## Discussion

A number of preclinical and clinical studies have reported an association between IL-4 levels and stroke pathology. For example, IL-4 expression increases transiently at early time points in the ischemic brain, which is attributed, at least partly, to its release from injured neurons or from infiltrated immune cells [19]. Cerebral IL-4 levels then return to baseline within 1 wk after stroke [16, 19]. In patients, serum levels of IL-4 are known to change following an acute stroke [33]. Furthermore, IL-4 gene polymorphisms are related to susceptibility to ischemic stroke and subsequent functional outcomes [34, 35]. Finally, a recent study reported the expression of IL-4R surrounding axonal filaments in postmortem brain tissue from patients with multiple sclerosis [31]. Although these clinical and experimental data are correlative, the present series of in vivo and in vitro studies firmly establish a causal link between IL-4 repletion and white matter integrity in preclinical stroke models. Deficiency of endogenous IL-4 exacerbated white matter disruption, whereas intranasal IL-4 treatment—even when delayed until 6 h post-stroke—improved the structural and functional integrity of white matter.

An improvement in white matter integrity might be achieved in two ways: preservation of preexisting white matter structures or enhancement of white matter repair. Our data favor the latter as the predominant mechanism of action for IL-4. First, longitudinal DTI revealed similar reductions in FA values shortly after stroke in vehicle or IL-4-treated mice, indicating comparable degrees of initial white matter injury during the early/acute phase. A significant improvement in white matter microstructure was only observed in IL-4-treated animals in later stages of recovery (28 and 35 d after stroke). These results strongly suggest a delayed effect of IL-4 on white matter repair, rather than preservation shortly after injury. Second, electron microscopy (EM) and histological studies revealed structural signs of remyelination in the ischemic brain, including thinner myelin segments than before stroke, and reduced paranode length with Caspr/Nav1.6 staining [36]. Each of these structural alterations were significantly improved by IL-4 treatment, indicating superior remyelination. Third, IL-4 treatment increased the number of new, mature oligodendrocytes (APC^+^BrdU^+^) in the ischemic brain 35 d after stroke. Such improvements in oligodendrogenesis are critical for successful remyelination. Finally, the LPC model of demyelination–remyelination unequivocally confirmed the white matter–specific effects of IL-4 in remyelination. As expected, the IL-4-mediated reconstitution of myelin structure culminated in functional improvements in neural conduction and behavioral performance, despite no changes in the degree of gray matter loss. Although IL-4 is important for white matter repair after brain injury, the IL-4 KO mice do not display deficits in myelin coverage in organotypic brain slice cultures under physiological conditions. This observation is consistent with the view that different mechanisms control myelin sheath formation during normal development and remyelination [36].

Although our T2 scanning did not show significant reductions in brain lesion size under conditions of IL-4 repletion, there was a trend toward lesion volume reduction at 14–35 d after stroke. As the IL-4 treatment was initiated 6 h after stroke, it remains possible that IL-4 may provide some degree of early protection [16]. It is also possible that some NeuN^+^ neurons remaining within the gray matter of mice without IL-4 might not be physiologically functional, although they are still histologically present, perhaps because their communication via white matter tracts is significantly impeded.

A previous study using mixed glial cultures demonstrated that the addition of IL-4 promotes the differentiation of OPCs into MBP-expressing oligodendrocytes in vitro, an effect mainly attributed to IL-4-mediated anti-inflammatory effects [37]. An additional conclusion emerging from our studies is that IL-4 enhances white matter repair at least partially independent of its well-established anti-inflammatory effects on microglia/macrophages. In IL-4-treated stroke mice, we observed an anti-inflammatory phenotypic change in microglia/macrophages, which is known to promote remyelination after CNS injury [28]. However, dramatic depletion of microglia/macrophages in the ischemic brain did not abolish the capacity of IL-4 to enhance oligodendrocyte replacement, supporting the involvement of microglia/macrophage-independent mechanisms. In vitro studies then confirmed that IL-4 can also directly target white matter components and promotes OPC differentiation into mature oligodendrocytes in the absence of microglia/macrophages. Previous fate mapping studies revealed that OPCs are responsible for generating new oligodendrocytes during remyelination [38, 39]. However, the differentiation of OPCs is frequently impaired in lesioned brains, mainly because of nonpermissive environmental cues [40–42]. We found that IL-4 treatment may overcome these obstacles for OPC differentiation in the ischemic brain, both directly by promoting OPC differentiation and indirectly through immunomodulation. Notably, endogenous oligodendrocyte differentiation has been observed until as late as 3 mo after CNS injuries [39], and this prolonged effort may provide a sufficiently wide therapeutic window that allows delayed IL-4 treatment to restore white matter and encourage long-term stroke recovery.

In the present study, we report a previously undefined mechanism of IL-4, namely that it directly induces OPC differentiation by activating PPARγ signaling. PPARγ is a master transcription factor known to promote OPC differentiation and maturation though manifold mechanisms involving enhanced myelin lipid synthesis, reduced oxidative stress, and promotion of mitochondrial function [43, 44]. Our previous work has shown that rosiglitazone, a PPARγ agonist, enhanced OPC proliferation and increased the number of newly generated mature oligodendrocytes after MCAO [45]. In addition, PPARγ may interact with histone deacetylases to modulate cell differentiation [46]. We demonstrated here that PPARγ inhibition abolished the effect of IL-4 on OPC differentiation in vitro and that OPC-specific KO of PPARγ in vivo reduced IL-4-enhanced oligodendrocyte replacement in the ischemic brain. These collective results indicate that IL-4-induced oligodendrocyte differentiation/maturation relies at least partially on the activation of PPARγ activity in OPCs. Precisely how IL-4/IL-4R engages PPARγ activity is not yet known, but several studies suggest that IL-4 may activate PPARγ in macrophages and other cell types through signal transducer and activator of transcription 6 (STAT6) signaling [37, 47]. Whether this pathway is also operational in OPCs lies beyond the scope of the present study but warrants further investigation.

Aside from the cell-specific effects of IL-4 on OPCs, we note that IL-4R is widely distributed in many types of CNS cells as well as infiltrated immune cells. Several lines of evidence from our study support the capacity of IL-4 to target multiple types of cells in addition to oligodendrocytes. First, we did not observe significant differences in the density of BrdU^+^APC^+^ cells between WT and PPARγ-OPC KO mice following MCAO, suggesting that endogenous IL-4 is able to promote white matter integrity by targeting other types of cells. Second, although PPARγ inhibition eliminated the effect of IL-4 on OPC differentiation in cultures, the absence of PPARγ in OPCs reduced, but did not abolish, the capacity of IL-4 to increase the number of newly generated oligodendrocytes in vivo. These results suggest that PPARγ expression in OPCs is an important but not exclusive mechanism for IL-4-induced oligodendrogenesis in preclinical stroke. Indeed, we observed that IL-4 treatment robustly promoted microglia/macrophage polarization toward an anti-inflammatory M2phenotype, as mentioned above. IL-4-stimulated microglia have been shown to release IL-4 induced 1 (IL4I1), which in turn augments remyelination [48]. Thus, direct and indirect effects on microglia/macrophages may contribute to the white matter repair afforded by IL-4. It has also been reported that the effects of IL-4 on adaptive immune responses contribute partially to recovery from neurotrauma [49]. In our hands, however, repeated intranasal IL-4 administration did not change T-lymphocyte subpopulations or B lymphocytes in blood and did not alter the composition of infiltrated lymphocytes. These data dispute the involvement of adaptive immune responses in IL-4-afforded neuroprotection in our stroke model.

Aside from its roles in immunoregulation, IL-4 can also directly target other types of CNS cells. For example, a recent study reported that the activation of IL-4R signaling in neurons may increase axonal outgrowth and ameliorate axonal pathology in a model of multiple sclerosis [31]. In this context, it is important to note that we did not observe improvements in the DTI measurement for axonal integrity (AD, λ_||_). In other disease models, multiple factors including inflammation, cellular infiltration, and gliosis may contribute to AD at chronic stages of disease. Therefore, the measurement of λ_||_ appears to lack sufficient resolution to distinguish axonal damage/repair versus confounding cellular responses over the course of progressive demyelination [50]. On the other hand, our EM data demonstrated an increase in axon diameter in IL-4-treated mice compared to vehicle-treated controls, supporting a beneficial role of IL-4 in maintaining axonal structure. These structural data were supported by our electrophysiological studies of axon conductance. Preservation of axonal structure and electrical function might provide an important feedback signal for OPC differentiation and guide remyelination [51, 52], preventing Wallerian degeneration of neurons after ischemia and enhancing white matter repair. In addition to these mechanisms, astrocytes are known to produce trophic factors in response to IL-4 stimulation, including brain-derived neurotrophic factor (BDNF) and nerve growth factor (NGF), both of which are also critical for brain function and brain repair [53].

A series of in vitro studies assessed the effective concentrations of IL-4 in various types of CNS cells. For example, IL-4 regulates microglia activity at concentrations of 1–20 ng/mL [19, 54]. IL-4 inhibits inflammatory responses and boosts trophic factor release in astrocytes at 5–10 ng/mL [53]. IL-4 protects neurons against excitotoxicity and stimulates axonal outgrowth in vitro at 50 ng/mL [31]. In the present study, IL-4 directly stimulated OPC differentiation within a concentration range of 5–100 ng/mL. These collective studies reveal similar effective IL-4 concentrations (in nanoscales) in glial and neuronal cells of the CNS. Indeed, it is precisely the multitargeting capacities of low-dose IL-4 that may render it an excellent therapy for CNS injuries characterized by a wide array of pathological mechanisms.

The ultimate implementation of preclinical research into clinical treatments requires extensive information on safety, optimal dose, route, and schedule of administration, among other variables. In the present study, IL-4 stimulated OPC differentiation in cultures at a concentration range of 5–100 ng/mL, and the optimal concentration was 10–20 ng/mL. Although there is neither a mathematical nor empirical method to accurately convert in vitro concentrations to in vivo intranasal dosage, these in vitro data support the capacity of IL-4 to directly target OPCs at low concentrations. Previous preclinical studies have similarly documented therapeutic effects of low doses of IL-4 in ischemic stroke and other neurological diseases. For example, subcutaneous IL-4 application at 2 μg/kg/d [19] or intracerebroventricular application of IL-4 at 60 ng/d [16] significantly improved long-term outcomes after stroke. Vogelaar and colleagues reported that intrathecal or intranasal (1 μg/d) IL-4 treatment ameliorated clinical signs and reversed disease progression in a model of multiple sclerosis [31]. Those studies, however, did not measure IL-4 concentrations in the brain after treatment. Here, we delivered IL-4 intranasally at a dose of 50 μg/kg/d (about 1.25 μg/d based on 0.025 kg average body weight), which significantly elevated IL-4 levels in key parts of the brain. This dose is similar to the effective intranasal dose for Vogelaar’s study and improved long-term functional recovery in our stroke model. Repeated dosing regimens or controlled continuous release was employed for all in vivo IL-4 treatment because of its rapid clearance and inactivation. The use of liposome-entrapped peptides has been reported to protect the cytokine and enhance its tissue delivery [55, 56]. Consistent with those findings, we demonstrated that IL-4 liposome nanoparticle delivery elevated IL-4 levels in the brain for at least 24 h. Further characterization of the optimal regimen of IL-4 treatment for stroke would accelerate its clinical translation.

Despite growing awareness of the importance of white matter restoration in poststroke rehabilitation, there is no effective therapy to preserve white matter function or to improve its repair in humans. Our study has uncovered a novel role of IL-4 in poststroke white matter recovery—a role that lies beyond its well-known immunoregulatory functions. Furthermore, we have shown that IL-4 promotes oligodendrogenesis and white matter repair in a PPARγ-dependent manner. Therefore, it seems reasonable to continue to test IL-4 as a therapeutic strategy for functional recovery after ischemic stroke. In particular, the intranasal route enables CNS IL-4 delivery, which is expected to significantly reduce the risk of peripheral side effects and expedite the eventual translation of IL-4 treatment for stroke victims.

## Materials and methods

### Ethics statement

All animal procedures were approved by the University of Pittsburgh Institutional Animal Care and Use Committee (approval number: 18032546), performed in accordance with the *National Institutes of Health Guide for the Care and Use of Laboratory Animals*, and reported in accordance with the *Animal Research*: *Reporting In Vivo Experiments* (ARRIVE) guidelines. All efforts were made to minimize animal suffering and the number of animals used. All animals were housed in a temperature- and humidity-controlled facility with a 12-h light/dark cycle. Food and water were available ad libitum.

### Experimental animals

C57BL/6J WT, IL-4 KO, PPARγ^flox/flox^, and PDGFRα^CreER^ mice were purchased from Jackson Laboratory (Bar Harbor, ME, United States). PDGFRα^CreER(+/−)^PPARγ^flox/flox^ mice were bred from PPARγ^flox/flox^ and PDGFRα^CreER^ mice. The depletion of PPARγ in OPCs was induced in PDGFRα^CreER(+/−)^PPARγ^flox/flox^ mice (8-wk-old males) by intraperitoneal injection of 4-hydroxytamoxifen (H7904, Sigma, St. Louis, MO, USA, 0.1 mg in 100 μL corn oil, daily for 5 consecutive days). For microglia/macrophage depletion, PLX5622 (Plexxikon, Berkeley, CA, USA) was supplied to mice (9–10 wk old, 25–30 g body weight) in the diet (Research Diets, New Brunswick, NJ, USA) at 1,200 PPM (1,200 mg/kg of chow), starting 7 d prior to surgery and continuing until the end of experiments.

### Murine models of transient cerebral ischemia

Transient cerebral ischemia was induced by intraluminal occlusion of the left middle cerebral artery (MCA) for 60 min, as described previously [57]. Sham-operated animals underwent the same anesthesia and exposure of arteries without MCAO. Briefly, mice were anesthetized with 3% isoflurane in a 30% O_2_/68.5% N_2_O mixture until they were unresponsive in the tail-pinch test. Mice were then fitted with a nose cone blowing 1.5% isoflurane in a 30% O_2_/68.5% N_2_O mixture under spontaneous breathing for anesthesia maintenance. An 8–0 monofilament with a silicon-coated tip was introduced into the common carotid artery, advanced to the origin of the MCA, and left in place to limit MCA blood flow for 60 min. Rectal temperature was controlled at 37.0 ± 0.5 °C using a temperature-regulated heating pad during surgery. Regional cerebral blood flow was measured in all stroke animals using laser Doppler flowmetry. Animals that did not display at least 70% reduction in regional cerebral blood flow during MCAO compared to pre-ischemia levels were excluded from further experimentation. Surgeries and quantification were performed by investigators blinded to animal genotypes and experimental grouping. Animals were randomly assigned to sham or cerebral ischemia groups and received randomized treatments using a lottery drawing box. A grand total of 287 mice (64 sham-operated and 223 ischemic mice) were used in this study, including 26 mice that were excluded from further assessments, either because of death after ischemia or failure of ischemia induction. The mortality during MCAO surgery was 7.6% in WT male mice (12/158), 14.3% in IL-4 KO male mice (1/7), 11.1% in microglia/macrophage-depleted mice (3/27), and 12.5% in PPARγ-OPC KO mice (3/24). There was no significant difference in mortality rate across groups.

### Intranasal IL-4 nanoparticle preparation and administration

Lecithin (150 mg) and cholesterol (15 mg) were dissolved in 5 mL of a chloroform–methanol mixed solution (3:1, volume:volume). A lipid layer was formed after evaporating the mixed solvent under vacuum distillation. Recombinant IL-4 protein (1 mg, Peprotech, Rocky Hill, NJ, USA) was dissolved in 5 mL of water and then added to the lipid layer for hydration for about 30 min. The mixture was homogenized by ultrasonic probes for 15 min in an ice bath and then filtered through a 0.45-μm membrane. The free IL-4 protein was removed by ultrafiltration with centrifugal filter units (100-KDa cutoff). The particle size and zeta potential of IL-4 protein-loaded liposomes were determined by dynamic light scattering (DLS). The concentration of IL-4 protein was determined by the bicinchoninic protein determination kit (Thermo Fisher Scientific, Pittsburgh, PA, USA).

Animals were randomly assigned to receive vehicle or IL-4 (50 μg/kg body weight, intranasal) nanoparticle treatment starting 6 h after MCAO and repeated at 1–7 d, 14 d, 21 d, and 28 d post-surgery. Briefly, under isoflurane anesthesia, five 2-μL drops (total 10 μL) of IL-4 nanoparticles were applied alternately into each nostril with a 2-min interval between drops. Control animals received an equal volume of vehicle control using the same anesthesia and delivery regimen.

### BrdU injections

In order to label proliferating cells, animals were intraperitoneally injected with the thymidine analogue BrdU (50 mg/kg, B9285, Sigma, St. Louis, MO, USA) twice a day (with an interval of at least 8 h) for 4 consecutive days, beginning at 3 d after MCAO.

### Behavioral tests

Behavioral tests were performed by an individual blinded to experimental groups. Sensorimotor functions were measured 3–35 d after MCAO.

#### The rotarod test

Briefly, mice were forced to run on a rotating drum (IITC Life Science, Woodland Hills, CA, USA) with speeds starting at 4 rpm and accelerating to 40 rpm within 400 s. Three consecutive trials were conducted for each mouse with an interval of 15 min. The time at which a mouse fell off the drum was recorded as the latency to fall. Data were expressed as mean values from three trials.

#### The adhesive removal test

The adhesive removal test was performed to assess tactile responses and sensorimotor asymmetries. Two 2 × 3-mm adhesive tapes were applied to lesioned forepaws. Tactile responses were measured by recording the time to initially contact the ipsilateral paws, as well as the time to remove the adhesive tape, with a maximum observation period of 120 s.

#### Morris water maze test

Cognitive function was analyzed with the Morris water maze test 23–28 d after MCAO, as described previously [16]. In the learning test, a square platform (11 × 11 cm^2^) was submerged 2 cm beneath the water surface in a circular pool (diameter = 109 cm) of opaque water. Mice were placed into the pool from one of the four locations and allowed to locate the hidden platform for 60 s. Each mouse was trained on 3 trials (with randomly assigned starting positions) per day to locate the platform for three consecutive days before MCAO. At the end of each trial, the mouse was placed on the platform or allowed to stay on the platform for 30 s with prominent spatial cues displayed around the room. The time spent to reach the platform (learning phase) was recorded. Three trials were performed on each day. The memory test was performed on day 28. The platform was removed, and a single 60-s probe trial was conducted. Time spent in the goal quadrant where the platform was previously located was recorded.

### Measurement of tissue loss and MBP loss

Animals were euthanized and perfused with saline followed by 4% PFA (Sigma-Aldrich; St. Louis, MO, USA) in PBS. Brains were cryoprotected in 30% sucrose in PBS. Coronal brain sections (25 μm) were sliced on a freezing microtome, and six equally spaced coronal brain sections encompassing the MCA territory were stained with a rabbit anti-NeuN antibody (ABN78, 1:500; Millipore, Burlington, MA, USA) or rabbit anti-MBP antibody (MBP ab40390, 1:500, Abcam, Cambridge, MA, USA). Loss of NeuN^+^ or MBP^+^ tissue was analyzed by a blinded observer using NIH Image J software. The infarct volume with edema correction was calculated as the volume of the contralateral hemisphere minus the noninfarcted volume of the ipsilateral hemisphere.

### Immunohistochemistry and image analysis

Coronal brain sections were subjected to immunofluorescence staining. Briefly, floating brain sections were blocked with 5% donkey serum in 0.3% Triton X-100 in PBS (PBST) for 1 h at room temperature, followed by overnight incubation with primary antibodies at 4 °C. After three washes in 0.3% PBST, sections were incubated with the appropriate secondary antibodies for 1 h at room temperature. The process was repeated once for double staining. Sections were then washed and mounted with DAPI Fluoromount-G or Fluoromount-G (Southern Biotech; Birmingham, AL, USA). For immunostaining using the mouse primary antibody, the M.O.M kit (BMK-2202, Vector Laboratories; Burlingame, CA, USA) was applied before primary antibodies to block nonspecific signals, according to the manufacturer’s instructions. Primary antibodies included goat anti-Iba1 (ab5076, Abcam, 1:500, Cambridge, MA, USA), mouse anti-CD206 (565250, 1:500, BD Bioscience, San Jose, CA, USA), rat anti-CD16 (553142, 1:500, BD Biosciences, San Jose, CA, USA), rat anti-BrdU (ab6326, 1:500, Abcam, Cambridge, MA, USA), rabbit anti-NG2 (AB5320, 1:400, Millipore, Billerica, MA, USA), mouse anti-APC (OP80, 1:500, Millipore, Billerica, MA, USA), mouse anti-Neurofilament H (NF-H) nonphosphorylated antibody (SMI32; 801701, 1:500, BioLegend, San Diego, CA, USA), mouse anti-Caspr antibody (MABN69, 1:300, Millipore, Billerica, MA, USA), and rabbit anti-Nav1.6 antibody (1:300, ASC-009 Alomone, Jerusalem, Israel). The following secondary antibodies (Jackson ImmunoResearch Laboratories; West Grove, PA, USA) were applied: anti-mouse secondary antibody conjugated with Cy3 (115-165-146), anti-rabbit secondary antibody conjugated with Cy3 (711-165-152), anti-rat secondary antibody conjugated with Cy3 (712-165-153), anti-rabbit secondary antibody conjugated with Alexa Flour 488 (711-545-152), anti-rat secondary antibody conjugated with Alexa Flour 488 (712-545-153), anti-goat secondary antibody conjugated with Alexa Flour 488 (705-545-147), and anti-mouse secondary antibody conjugated with Alexa Flour 488 (715-545-151). All the secondary antibodies were diluted 1:1,000. Confocal microscopy (FluoView FV1000; Olympus, Tokyo, Japan) was used to capture images.

Image analyses were performed on one or two randomly selected microscopic fields in the peri-infarct areas of the EC, two in the cortex, and two in the striatum of each section; two sections covering the infarct area were assessed for each mouse brain. The recorded images were loaded onto Image J (NIH) and were manually quantified by 2 observers blinded to grouping. Positively stained cells were electronically labeled with the software to avoid duplicated counting. The infarct area was identified as the region in which the majority of DAPI-stained nuclei were shrunken. The border of the infarct was determined by the loss of NeuN staining and the accumulation of Iba1^+^ microglia/macrophage or BrdU^+^APC^+^ oligodendrocytes on adjacent sections. We defined the peri-infarct area as the tissue that covers a radial distance of 200–300 μm from the border of the infarct.

### Flow cytometry and ImageStream

Animals were euthanized and perfused with cold saline. Brains were dissected, and the ipsilateral (left) and contralateral (right) hemispheres were collected. Brains were dissociated into a single-cell suspension using a gentleMACS Dissociator (Miltenyl Biotec) following the manufacturer’s instructions. The suspension was passed through a 70-μm cell strainer (Fisher Scientific, Pittsburgh, PA, USA) and resuspended in 30% Percoll (GE Health). Single-cell suspensions were separated from myelin and debris by centrifugation (500*g*, 30 min, 18 °C) on a 30%–70% Percoll gradient. Cells at the interface were collected and washed with Hank’s balanced salt solution (HBSS; Sigma-Aldrich, St. Louis, MO, USA) containing 1% fetal bovine serum (Sigma-Aldrich, St. Louis, MO, USA) and 2 mM EDTA (Sigma-Aldrich, St. Louis, MO, USA) before staining. Blood cells were prepared as described previously [57]. Single-cell samples were first incubated with antibodies to surface antigens for 30 min on ice at 4 °C in the dark. After two washes, cells were fixed and permeabilized with an intracellular staining kit (Thermo Fisher eBioscience, Pittsburgh, PA, USA) according to the manufacturer’s protocol. The antibodies used were as follows: anti-CD45-FITC (Biolegend, 103108, 1:400; San Diego, CA, USA), anti-GLAST-APC (Miltenyi Biotec, 130-098-803, 1:10; Auburn, CA, USA), anti-PDGFRα-PE-Cy7 (Biolegend, 135912, 1:400), anti-O4-APC (Miltenyi Biotec 130-109-155, 1:50), anti-CD11b-PE-Cy7 (Biolegend, 10126, 1:100), anti-NeuN (Abcam, ab209898, 1:100, Cambridge, MA, USA), anti-rabbit IgG BV421 (Biolegend, 406410, 1:100; San Diego, CA, USA), anti-IL-4Rα PE (R & D Systems, FAB530P, 1:20; Eugene, OR, USA), anti-GFAP-AF-488 (BioLegend, 644704; 1:400, San Diego, CA, USA), and anti-A_2_B_5_ (R & D Systems, FAB1416U, 1:20, Eugene, OR, USA). For lymphocyte subpopulation induction, blood or brain cells were stimulated with cell stimulation cocktail (ebioscience, 00-4975-03; Grand Island, NY, USA) for 6 h and stained with fluorophore-labeled antibodies (CD4, CD3, CD8, CD19, Foxp3, IL-4, IL-17, and IFN-γ). Appropriate isotype controls were used according to the manufacturer’s instructions (Thermo Fisher eBioscience, Pittsburgh, PA, USA). Fluorochrome compensation was performed with single-stained OneComp eBeads (Thermo Fisher eBioscience, Pittsburgh, PA, USA). Flow cytometry was performed on the BD LSRII flow cytometer (BD Biosciences). Data analyses were performed using FlowJo software (FlowJo, version 10.0, Ashland, OR, USA). ImageStream analysis was performed using an Amnis Imaging Flow Cytometer (Millipore). Six thousand cells were collected in each sample. Data analyses were performed using IDEAS software (Millipore).

### CAP measurements

CAPs were measured in the EC. Mice were decapitated after CO_2_ euthanasia, and brains were removed. Coronal slices (350 μm thick) were prepared −1.06 mm from bregma and transferred to an incubation chamber containing pregassed (95% O_2_/5% CO_2_) artificial cerebrospinal fluid (aCSF; 126 mmol/L NaCl, 2.5 mmol/L KCl, 1 mmol/L Na_2_H_2_PO_4_, 2.5 mmol/L CaCl_2_, 26 mmol/L NaHCO_3_, 1.3 mmol/L MgCl_2_, 10 mol/L glucose [pH 7.4]) for 30 min at 34 °C and then for 1 h at room temperature (25 °C). Brain slices were then perfused with aCSF at a constant rate (3–4 mL/min) at 25 °C. For recording, a bipolar stimulating electrode (intertip distance, 100 μm) was placed across the CC at about 0.9 mm lateral to the midline. A glass extracellular recording pipette (5–8 MΩ tip resistance when filled with aCSF) was placed in the EC, 0.25, 0.5, 0.75, and 1 mm lateral from the stimulating electrode. The recording at 0.75 mm from the stimulating electrode was reported. The CAP signal was digitized (Digidata 1500B plus humsilencer; Molecular Devices, San Jose, CA, USA), amplified (×1 K), and recorded by the Axoclamp 700B amplifier (Molecular Devices, San Jose, CA, USA). The signal was then analyzed by pClamp 10 software (Molecular Devices, San Jose, CA, USA). The input–output curves were generated by increasing the stimulating intensity by 0.25-mA intervals from baseline, up to 2 mA. The amplitude of the N1 component of the CAP (representing myelinated fibers) was calculated as the variance from the first peak to the first trough.

### Transmission electron microscopy

TEM was used to measure myelin thickness in the CC/EC area. Mice were perfused with ice-cold saline, followed by 4% PFA and 2.5% glutaraldehyde in 0.1 mol/L PBS buffer. The CC/EC tissue near the site of ischemia was microdissected into 1-mm^3^ blocks. These specimens were immersion-fixed for 24 h in 2% glutaraldehyde. Tissue was then rinsed in PBS and fixed in 1% osmium tetroxide in 0.1 M PBS for 45 min. Samples were dehydrated in increasing concentrations of acetone and embedded in Araldite resin. Ultrathin 60-nm sections were cut on a Leica UCT ultramicrotome with a diamond knife (Diatome, Wetzlar, Germany), stained with uranyl acetate and lead citrate, and viewed using a JEM1400 TEM (JEOL, Akishima, Japan). Two sections from each animal were analyzed. Three to 5 images (600 μm^2^ each) were acquired in randomly selected areas within the CC/EC from each section at a magnification of 50,000× and analyzed with Image J by an investigator blinded to experimental groups. At least 50 random axons per animal were analyzed by tracing the axonal circumference and the whole fiber circumference in a blinded fashion. G-ratios were calculated as the ratio of the inner axonal diameter to the total outer diameter (axonal diameter + total myelin sheath thickness).

### MRI

For in vivo MRI, mice were initially anesthetized with 3% isoflurane and then positioned on an animal cradle with a stereotaxic head holder. Anesthesia was maintained between 1% and 1.5% isoflurane throughout the experiment. Respiration and temperature were monitored continuously, and temperature was maintained by a warm animal heating system (SA Instruments, Stony Brook, NY, USA). MRI was performed on a 9.4 T/30-cm AVIII HD spectrometer (Bruker Biospin, Billerica, MA, USA) equipped with a 12-cm high-performance gradient set and using an 86-mm quadrature RF transmit volume coil and a 2-channel receive surface RF coil and Paravision 6.0.1. A T_2_-weighted RARE sequence was used to visualize the infarct, with the following setup: repetition time (TR)/echo time (TE) = 4,000/40 ms, field of view (FOV) = 20 × 20 mm, acquisition matrix = 256 × 256, 16 slices with a slice thickness of 0.5 mm, 4 averages, and a RARE factor = 8. The DTI data were collected using an echo planar imaging (EPI)-DTI imaging sequence with the same geometry as the T_2_-weighted scan, using the following setup: TR/TE = 2,300/22 ms, FOV = 20 × 20 mm, acquisition matrix = 128 × 128, 16 slices with a slice thickness of 0.5 mm, 8 averages, 2 segments, 5 A0 images and 30 noncolinear diffusion images, Δ/δ = 10/3 ms, and a b-value = 1,000 s/mm^2^.

Perfusion-fixed brains were left in the skull and imaged ex vivo using a Bruker AV3HD 11.7 T/89-mm vertical-bore microimaging system equipped with a Micro2.5 gradient set capable of 1,500 mT/m, a 20-mm quadrature RF resonator, and Paravision 6.0.1 (Bruker Biospin, Billerica, MA, USA). DTI data were collected using a multislice spin-echo sequence with 5 A0 images and 30 noncolinear diffusion images with the following setup: TE/TR 22/2,800 ms, 2 averages, 160 × 160 matrix, 16 × 16-mm FOV, 25 slices, 0.5 mm slice thickness, b-value = 3,000 s/mm^2^, and Δ/δ = 11.0/5.0 ms. This setup has been established for the acquisition of high-resolution DTI scanning in small animals [8, 58, 59].

DTI data were analyzed with DSI Studio software (http://dsi-studio.labsolver.org/). ROIs were drawn manually in a blinded manner encompassing the EC and IC in the ipsilesional and contralesional hemispheres to determine FA, AD, and RD. DEC, FA, and RD maps were generated by DSI Studio software.

### PCR

In order to verify cre-induced deletion of flanked PPARγ exons, a pair of primers (forward: 5′-TGTAATGGAAGGGCAAAAGG; reverse: 5′-TGGCTTCCAGTGCATAAGTT) that flank the downstream loxp site were used to perform PCR with genomic DNA. Program for PCR was 98 °C for 1 min; (98 °C for 15 s; 60 °C for 15 s; 72 °C for 15 s) × 38, and then 72 °C for 1 min. A pair of primers that detect the Cre cDNA (forward: 5′-GCGGTCTGGCAGTAAAAACTATC; reverse: 5′-GTGAAACAGCATTGCTGTCACTT) were used as a positive control to confirm the absence of PCR band in OPC was not due to failed PCR protocol itself.

### Microarray

Microarray experiment was performed in the Genomics Research Core in the University of Pittsburgh. Total RNA was prepared from IL-4- or PBS-treated OPCs using Trizol (Qiagen) according to manufacturer’s instruction. Preparation of cDNA was performed using the WT Plus reagent kit (Thermo Fisher) according to the manufacturer’s instructions. Reverse transcription was performed using 100 ng total RNA and dNTP-T7 random primers. Second-strand synthesis and in vitro transcription created amplified cRNA. cDNA from a second reverse transcription reaction was fragmented and end-labeled with biotin for hybridization to the Affymetrix Clariom S rat array. Following overnight (16 h) hybridization at 45 °C with rotational mixing at 60 rpm, arrays were processed on a GeneChip 450 Fluidics Station using manufacturer-specified protocols. To remove unbound sample, arrays were first washed with nonstringent wash buffer A. The GeneChips were then stained for 10 min in stain cocktail 1. Buffer A was again used to wash off excess stain. Signal amplification was achieved by 10-min incubation with stain cocktail 2 followed by a second 10-min incubation with stain cocktail 1. The chip was washed with high-stringency buffer B and filled with Array Holding Buffer before being removed from the fluidics station and scanned using the GeneArray 3000 scanner. First-level image analysis was performed using Affymetrix Expression Console. Four biologic replicates were performed for each experimental condition. Data resulted in 23,188 detected probes that were used for subsequent analysis using Transcriptome Analysis Console (Affymetrix) and Metascape analysis [32] (http://metascape.org). The GO network plot is visualized using Cytoscape, in which terms with a similarity > 0.3 are connected by edges. Each node represents an enriched term and is colored by its cluster ID.

### Primary OPC and oligodendrocyte cultures

Primary OPC cultures were isolated with the differential attachment method. Briefly, brains of mixed-sex Sprague Dawley rat pups (postnatal day 1–2) were triturated after removing the meninges and blood vessels. The brain tissues were dissociated with Trypsin (0.01%) at 37 °C for 15 min. After washing with ice-cold DMEM, the cells were passed through a 70-μm filter and plated onto poly-D-lysine-coated T-flasks filled with culture media (DMEM/F12 containing 10% heat-inactivated fetal bovine serum, 2 mM l-glutamine, 1 mM sodium pyruvate, 100 μM nonessential amino acids, 50 U/mL penicillin, and 50 μg/mL streptomycin) [45]. Cells were grown to confluence (12–14 d in vitro [DIVs]) in a humidified incubator at 37 °C and with 5% CO_2_. Microglia were removed by shaking the mixed glia–containing flasks for 1 h at 180 rpm. After removing microglia, flasks were subjected to shaking at 200 rpm overnight to separate OPCs from the astrocyte layer. OPCs were maintained for 3–5 d in a serum-free basal defined medium (BDM: DMEM, 0.1% bovine serum albumin, 50 μg/mL human apo-transferrin, 50 μg/mL insulin, 30 nM sodium selenite, 10 nM D-biotin, 10 nM hydrocortisone) containing 10 ng/mL PDGF and 10 ng/mL bFGF. For oligodendrocyte induction, OPCs were stimulated with T3 and CNTF (10 ng/mL). The medium was exchanged every 2 d.

### Western blot

Protein was isolated from in vitro cultures. Western blots were performed using the standard SDS-polyacrylamide gel electrophoresis (PAGE) method. The primary antibodies used in this study include rabbit anti-MBP (Cell signaling Technology, 78896, 1:500, Danvers, MA, USA), mouse MOG (Santa Cruz, Sc-166172, 1:200, Santa Cruz, CA, USA), and mouse anti-β-actin (A2228, 1:20,000; Sigma-Aldrich, St. Louis, MO, USA). In brief, polyvinylidene difluoride (PVDF) membranes were blocked in 5% nonfat milk for 1 h at room temperature and then incubated at 4 °C overnight with primary antibodies. Next, the membrane was incubated with secondary antibodies for 1 h at room temperature (1:10,000, LI-COR Biosciences, Lincoln, NE, USA), and scanned with LI-COR Odyssey Infrared Imaging System 9201-550U (LI-COR Biotechnology, Lincoln, NE, USA). ImageJ software was used for gel analyses.

### PPAR activity assay

Nuclear extracts were prepared from OPCs using NE-PE Nuclear and Cytoplasmic Extraction Reagent Kit (Thermo Fisher, 78833, Pittsburgh, PA, USA). PPAR activity was assessed using the PPAR (α, δ, γ) Transcription Factor Assay Kit (Abcam, ab133113, Toronto, ON, Canada) or the PPARγ Transcription Factor Assay Kit (Cayman Chemical, 10006855, Ann Arbor, MI, USA) following the manufacturers’ instructions.

### Organotypic culture preparation, treatment, and immunostaining

The cerebellum and attached hindbrain were isolated from P9 C57BL/6J mouse pups, sectioned sagittally at 300 μm on a McIlwain tissue chopper, and plated onto Millipore-Millicel-CM mesh inserts (Thermo Fisher Scientific, Pittsburgh, PA, USA) in 6-well culture plates with media containing 50% basal medium Eagle (Sigma, St. Louis, MO, USA, B1522), 25% heat-inactivated horse serum (GIBCO 26050–070), 25% Earle’s balanced salt solution (GIBCO 24020–117), 6.5 mg/mL glucose (Sigma G8769), 1% penicillin-streptomycin, and 1% Glutamax. Media were changed every 2–3 d. To induce demyelination, cultures were treated with LPC (Sigma, 0.5 mg/mL) for 17 h. Slices were washed in media for 10 min and treated for 3 or 7 d with PBS or IL-4 (20 ng/mL, IL-4 was added to fresh media every other day). Slices were fixed in 4% PFA for 1 h. Rabbit anti-MBP antibodies (Santa Cruz, Santa Cruz, CA, USA, sc-13914; 1:200) and mouse anti-NF-H antibodies (Abcam, Cambridge, MA, USA, ab8135, 1:1,000) were applied for 48 h at 4 °C. Fluorescence-conjugated secondary antibodies were applied overnight at 4 °C. Slices were mounted onto glass slides and coverslipped with Fluoromount-G. Confocal microscopy (Fluoview FV1000; Olympus, Tokyo, Japan) was used to capture images.

### Myelination assessment and quantification on organotypic cultures

MBP staining intensity was analyzed with Imaris software (Bitplane AG). Morphology reconstructions were carried out with the Imaris FilamentTracer module. Quantitative assessment of myelination was performed in a blinded manner using the Imaris colocalization module. Thresholds for colocalization of red (NFH^+^ axon) and green (MBP^+^ myelin) channels were calculated automatically and maintained across different images from the same batch of staining. A colocalization channel was created and channel statistics were calculated automatically. The myelination index was calculated as the percent of colocalized areas in the total NFH^+^ red pixels above threshold. For each condition, at least 6 slices collected from three independent experiments were analyzed. Two to three fields were imaged from each slice.

### Statistical analysis

Sample sizes for animal studies were determined by power analyses based on pilot studies or the literature. Results are presented as mean ± standard error of the mean (SEM). GraphPad Prism software (version 8.1.0, La Jolla, CA, USA) was used for statistical analyses. The Student’s *t* test was used for comparison of two groups for continuous variables with normal distributions. The Mann-Whitney U rank sum test was used for continuous variables with nonnormal distributions. The differences in means among multiple groups were analyzed using one-way ANOVA. Differences in means across groups with repeated measurements over time were analyzed using the repeated-measures ANOVA. When the ANOVA showed significant differences, pairwise comparisons between means were tested by post hoc Bonferroni tests. The correlation analyses between continuous data with normal distribution were performed using Pearson correlation analysis. Spearman rank correlation analyses were used to test correlations between data sets with nonnormal distributions. In all analyses, *p* < 0.05 was considered statistically significant.

## Supporting information

S1 Fig IL-4Rα expression on oligodendrocyte lineage cells. ImageStream images demonstrate the expression of IL-4Rα on O4^+^ preoligodendrocytes and premyelinating oligodendrocytes (A), A_2_B_5_^+^ OPCs (B), and CD11b^+^ microglia/macrophages (C) 3 d after stroke. IL-4Rα, interleukin-4 receptor α; OPC, oligodendrocyte progenitor cell.
(TIF)

S2 Fig The impact of IL-4 treatment on poststroke white matter integrity. Transient focal ischemia was induced by 60-min MCAO. Stroke mice were post-treated with IL-4 or vehicle starting from 6 h after MCAO at daily intervals for days 1–7 and then every 7 d until 28 d after stroke. (A) Representative axial views (5 sections from rostral to caudal) of DEC maps 35 d after stroke. Arrows indicate the EC. Arrow heads indicate the IC. (B) In ex vivo DTI, the ipsilateral fiber volume was markedly lower than the fiber volume on the contralateral side on day 35 after stroke, indicating white matter fiber loss at that time point. Arrow points to lesioned hemisphere with white matter loss. (C, D) Quantification of AD values in the EC (C) and IC (D) at five axial levels from rostral to caudal. *n* = 6/group. Data are expressed as the ratio of AD value in the ipsilateral (lesioned) side to the AD value in the nonlesioned contralateral hemispheres. Data associated with this figure can be found in the supplemental data file (S1 Data). AD, axial diffusivity; EC, external capsule; IC, internal capsule; IL-4, interleukin-4; DTI, diffusion tensor imaging; MCAO, middle cerebral artery occlusion.
(TIF)

S3 Fig APC is not expressed in GFAP^+^ astrocytes in the ischemic brain. Brain slices collected 35 d after 60-min MCAO were stained for astrocyte marker GFAP (red) and oligodendrocyte marker APC (green). Nuclear staining with DAPI was shown in blue. Scale bar: 40 μm. APC, adenomatous polyposis cell; GFAP, glial fibrillary acidic protein; MCAO, middle cerebral artery occlusion.
(TIF)

S4 Fig IL-4 treatment shifts microglia/macrophage polarization toward an anti-inflammatory phenotype. Brains were collected 7 d and 14 d after 60-min MCAO. Brain slices were stained for microglia/macrophage marker Iba1 and anti-inflammatory phenotype marker CD206 or proinflammatory phenotype marker CD16. (A-B) Quantification of CD206^+^Iba1^+^ anti-inflammatory microglia/macrophages in the peri-infarct areas in CTX and STR at 7 d (A) and 14 d (B) after MCAO. (C-D) Quantification of CD16^+^Iba1^+^ pro-inflammatory microglia/macrophages in the peri-infarct areas in CTX and STR at 7 d (C) and 14 d (D) after MCAO. *n* = 3–5 mice per group. *p < 0.05, **p < 0.01, ***p < 0.001. One-way ANOVA and Bonferroni post hoc tests. Data associated with this figure can be found in the supplemental data file (S1 Data). CTX, cortex; Iba1, ionized calcium binding adaptor molecule 1; IL-4, interleukin-4; MCAO, middle cerebral artery occlusion; STR, striatum.
(TIF)

S5 Fig PLX5622 depletes phagocytes under physiological conditions and after stroke injury. For microglia/macrophage depletion, PLX5622 was supplied in the diet (1,200 mg/kg of chow) to normal mice or stroke mice starting 7 d prior to 60-min MCAO and continued until sacrifice. (A-B) Representative images of double staining for Iba1 (green) and NeuN (red). Scale bar: 50 μm. (C-D) Quantification of Iba1^+^ microglia (C) and NeuN^+^ neurons (D) in sham brains 7 or 28 d after initiation of the PLX5622 diet. *n* = 3/group. (E-F) Quantification of Iba1^+^ microglia/macrophages (E) and NeuN^+^ neurons (F) in ischemic brains 28 d after initiation of the PLX5622 diet (21 d after stroke). *n* = 3/group. (G-H) Flow cytometry of the immune cells in the blood 28 d after initiation of the PLX5622 diet (21 d after stroke). Data are expressed as % of single cells. *n* = 6/group. **p < 0.01; ***p < 0.001. One-way ANOVA and Bonferroni post hoc test (C and D) or Student’s *t* test (E, F, and H). Data associated with this figure can be found in the supplemental data file (S1 Data). Iba1, ionized calcium binding adaptor molecule 1; MCAO, middle cerebral artery occlusion.
(TIF)

S6 Fig Intranasal IL-4 treatment exerts minimal effects on adaptive immune cell populations. Transient focal ischemia was induced by 60-min MCAO. Stroke mice were post-treated with IL-4 or vehicle starting 6 h after MCAO at daily intervals for days 1–7. Animals were sacrificed on day 8 after stroke. (A) Gating strategy for blood lymphocyte populations. (B) Histograms showing the IL-4^+^ Th2 cell, IL-17^+^ Th17 cell, regulatory T cell (Foxp3^+^), and IFNγ^+^ Th cell subpopulations in CD4^+^ T cells in the blood of vehicle or IL-4-treated mice. (C) Quantification of lymphocyte populations in the blood. (D) Quantification of lymphocyte populations in ischemic brains. *n* = 6/group. Student’s *t* test. Data associated with this figure can be found in the supplemental data file (S1 Data). Foxp3, forkhead box P3; IFNγ, interferon gamma; IL-4, interleukin-4; MCAO, middle cerebral artery occlusion; Th, T helper.
(TIF)

S7 Fig IL-4 exerts no effect on OPC survival and proliferation. OPCs were treated with vehicle and various concentrations of IL-4 for 3 d. (A) LDH assay for cell death. (B) MTT assay. (C) Quantification of OPC proliferation using BrdU proliferation kit (Sigma-Aldrich). Three independent experiments, each performed in quadruplicate. Data associated with this figure can be found in the supplemental data file (S1 Data). BrdU, 5-bromo-2′-deoxyuridine; IL-4, interleukin-4; LDH, lactate dehydrogenase; OPC, oligodendrocyte progenitor cell.
(TIF)

S8 Fig GO terms that were significantly enriched in up-regulated (A) or down-regulated (B) DEGs in IL-4-treated OPCs. Microarray analyses were performed on OPCs treated with PBS or IL-4 (20 ng/mL) for 3 d. *n* = 4/group. DEGs that were up-regulated or down-regulated (FDR < 0.05; and fold change > 2) by IL-4 treatment were subjected to GO enrichment analysis using the Metascape analysis. The top 20 enriched GO terms in three categories (biological process, cellular component, and molecular function) are listed. Bars represent gene counts. Circles represent values of −logP. DEG, differentially expressed gene; FDR, false discovery rate; GO, gene ontology; OPC, oligodendrocyte progenitor cell.
(TIF)

S9 Fig Full size western blot images of MOG, MBP, and Actin in Fig 8. MBP, myelin basic protein; MOG, myelin oligodendrocyte glycoprotein.
(TIF)

S10 Fig OPC-specific PPARγ KO shows no impact on IL-4R expression on immature oligodendrocytes. Brain slices were collected from WT or OPC-specific PPARγ KO mice 35 d after 60-min MCAO. Brain slices were stained for O4 (green)—a marker of preoligodendrocytes and premyelinating oligodendrocytes—and IL-4R (red). Yellow arrows indicate double-stained cells. Scale bar: 40 μm. IL-4R, interleukin-4 receptor; KO, knockout; MCAO, middle cerebral artery occlusion; OPC, oligodendrocyte progenitor cell; PPARγ, peroxisome proliferator-activated receptor gamma; WT, wild-type.
(TIF)

S1 Table DEGs up-regulated in oligodendrocyte progenitor cells by IL-4 treatment. DEG, differentially expressed gene; IL-4, interleukin-4.
(XLSX)

S2 Table DEGs down-regulated in oligodendrocyte progenitor cells by IL-4 treatment. DEG, differentially expressed gene; IL-4, interleukin-4.
(XLSX)

S1 Data Raw data. (XLSX)

## Abbreviations

AD: axial diffusivity
APC: adenomatous polyposis coli
BDNF: brain-derived neurotrophic factor
BrdU: 5-bromo-2′-deoxyuridine
CAP: compound action potential
Caspr: contactin-associated protein
CC: corpus callosum
CNS: central nervous system
DEG: differentially expressed gene
DTI: diffusion tensor imaging
EC: external capsule
EM: electron microscopy
FA: fractional anisotropy
FDR: false discovery rate
FSC-W: forward scatter width
GalC: galactocerebroside
GFAP: glial fibrillary acidic protein
GO: gene ontology
IC: internal capsule
IL-4: interleukin-4
IL4I1: IL-4 induced 1
IL-4RAb: IL-4 receptor α neutralizing antibody
IL-4Rα: IL-4 receptor α
KEGG: Kyoto Encyclopedia of Genes and Genomes
KO: knockout
LPC: lysophosphatidylcholine/lysolecithin
LSD: least significant difference
MBP: myelin basic protein
MCAO: middle cerebral artery occlusion
MFI: mean fluorescence intensity
MOG: myelin oligodendrocyte glycoprotein
MRI: magnetic resonance imaging
NFH: neurofilament-heavy
NG2: neuron-glia antigen 2
NGF: nerve growth factor
NOR: nodes of Ranvier
OPC: oligodendrocyte progenitor cell
PCA: principal component analysis
PI3K: phosphoinositide 3-kinase
PPARγ: peroxisome proliferator-activated receptor gamma
qRT-PCR: quantitative reverse transcription PCR
RD: radial diffusivity
SSC-A: side scatter area
STAT6: signal transducer and activator of transcription 6
TEM: transmission electron microscopy
WT: wild-type
