# Supplementary figures and images for: The interleukin-4/PPARγ signaling axis promotes oligodendrocyte differentiation and remyelination after brain injury
Source: PLoS Biol. 2019 Jun 21;17(6):e3000330. doi: 10.1371/journal.pbio.3000330 (PMC6608986; doi:10.1371/journal.pbio.3000330)

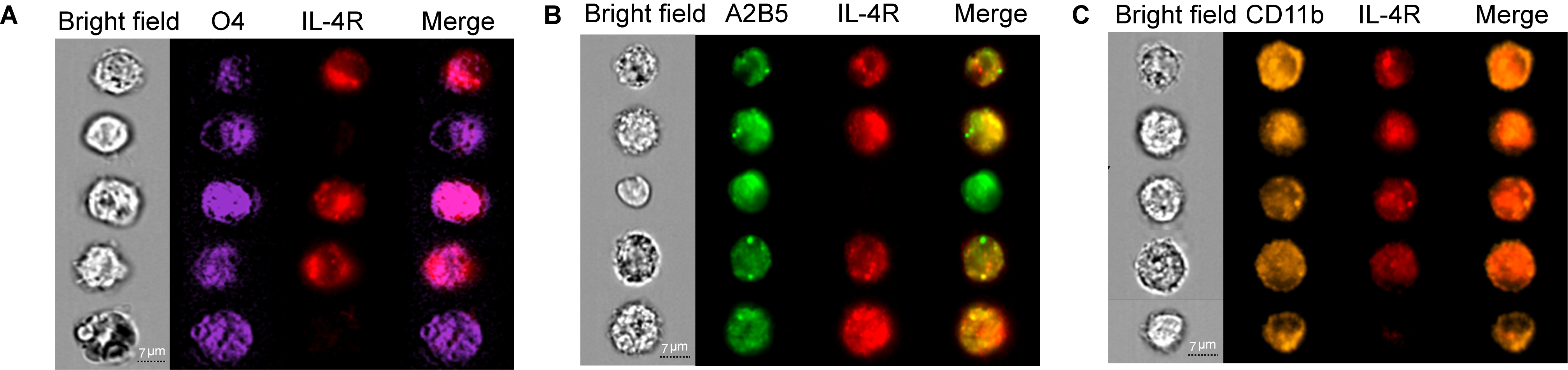

Supplement: S1 Fig — ImageStream images demonstrate the expression of IL-4Rα on O4+ preoligodendrocytes and premyelinating oligodendrocytes (A), A2B5+ OPCs (B), and CD11b+ microglia/macrophages (C) 3 d after stroke. IL-4Rα, interleukin-4 receptor α; OPC, oligodendrocyte progenitor cell. (TIF) [file pbio.3000330.s001.tif]

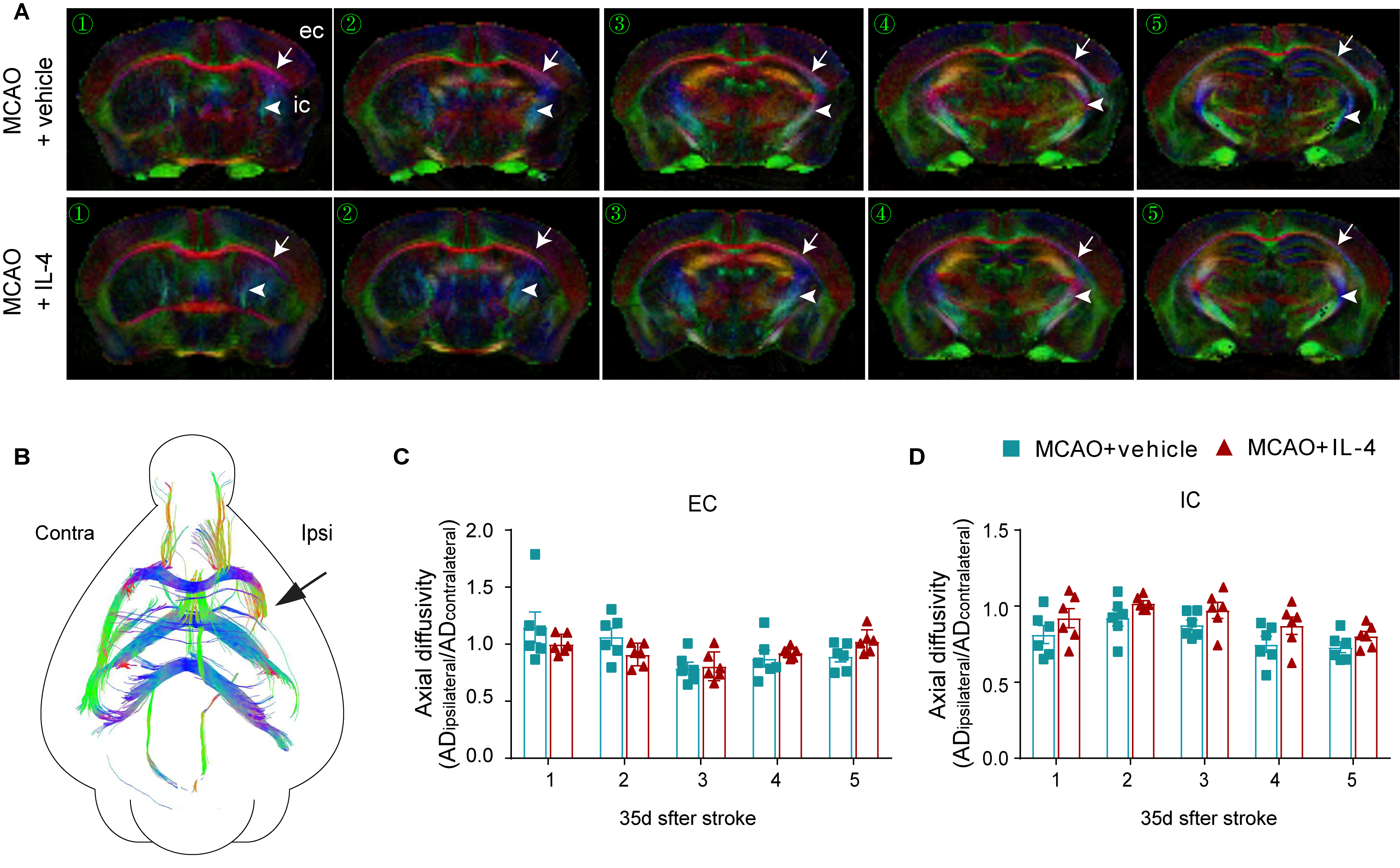

Supplement: S2 Fig — Transient focal ischemia was induced by 60-min MCAO. Stroke mice were post-treated with IL-4 or vehicle starting from 6 h after MCAO at daily intervals for days 1–7 and then every 7 d until 28 d after stroke. (A) Representative axial views (5 sections from rostral to caudal) of DEC maps 35 d after stroke. Arrows indicate the EC. Arrow heads indicate the IC. (B) In ex vivo DTI, the ipsilateral fiber volume was markedly lower than the fiber volume on the contralateral side on day 35 after stroke, indicating white matter fiber loss at that time point. Arrow points to lesioned hemisphere with white matter loss. (C, D) Quantification of AD values in the EC (C) and IC (D) at five axial levels from rostral to caudal. n = 6/group. Data are expressed as the ratio of AD value in the ipsilateral (lesioned) side to the AD value in the nonlesioned contralateral hemispheres. Data associated with this figure can be found in the supplemental data file (S1 Data). AD, axial diffusivity; EC, external capsule; IC, internal capsule; IL-4, interleukin-4; DTI, diffusion tensor imaging; MCAO, middle cerebral artery occlusion. (TIF) [file pbio.3000330.s002.tif]

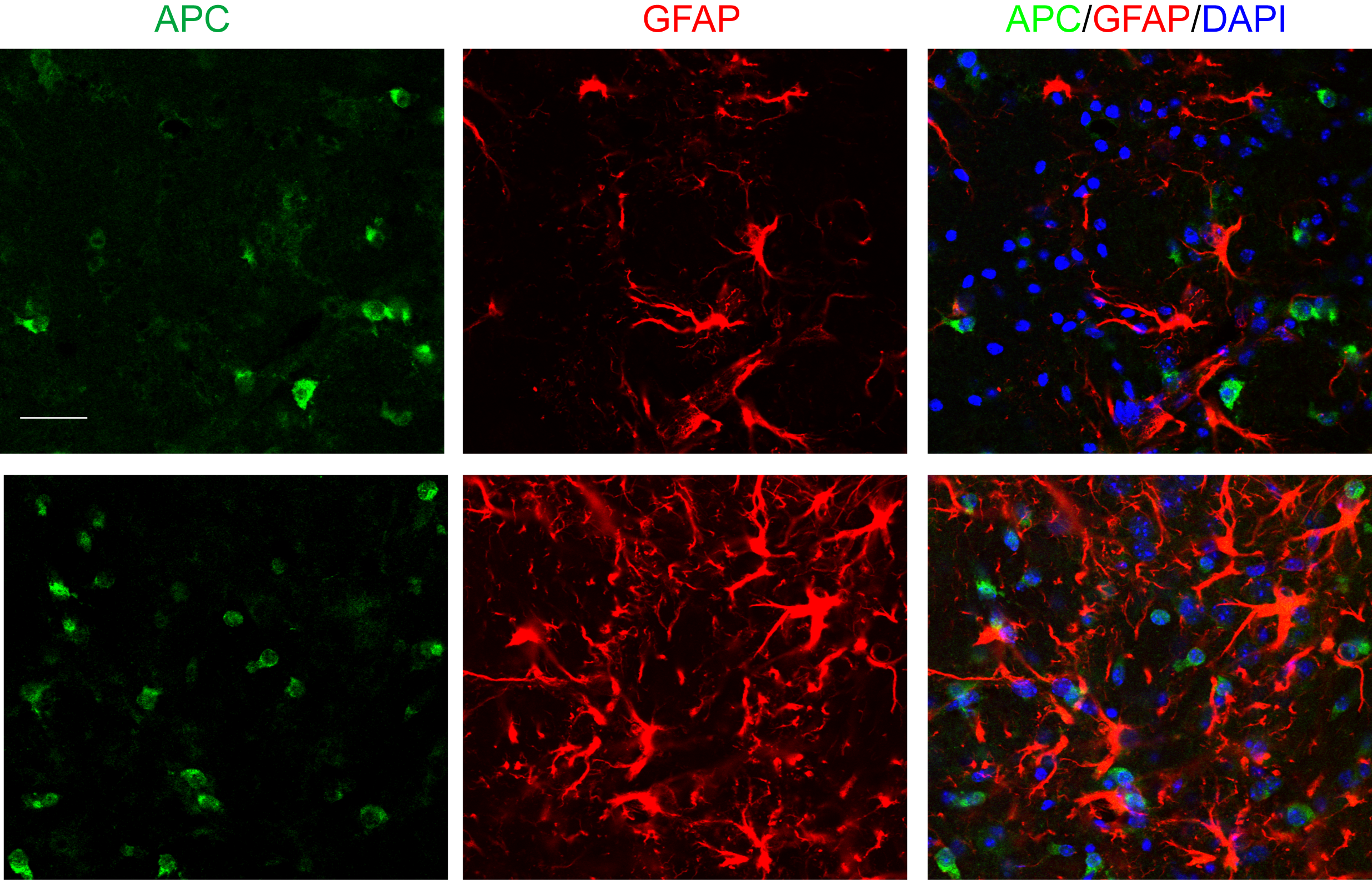

Supplement: S3 Fig — Brain slices collected 35 d after 60-min MCAO were stained for astrocyte marker GFAP (red) and oligodendrocyte marker APC (green). Nuclear staining with DAPI was shown in blue. Scale bar: 40 μm. APC, adenomatous polyposis cell; GFAP, glial fibrillary acidic protein; MCAO, middle cerebral artery occlusion. (TIF) [file pbio.3000330.s003.tif]

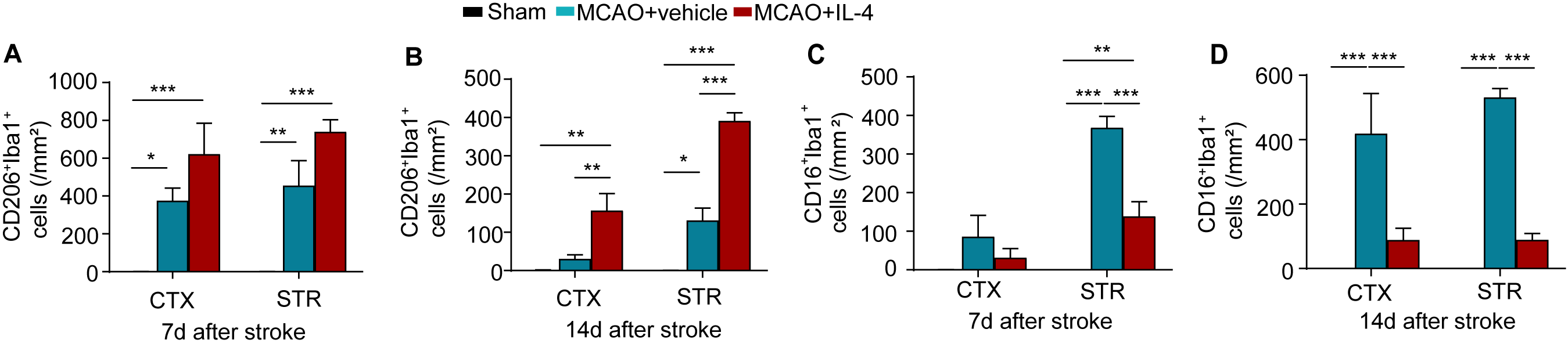

Supplement: S4 Fig — Brains were collected 7 d and 14 d after 60-min MCAO. Brain slices were stained for microglia/macrophage marker Iba1 and anti-inflammatory phenotype marker CD206 or proinflammatory phenotype marker CD16. (A-B) Quantification of CD206+Iba1+ anti-inflammatory microglia/macrophages in the peri-infarct areas in CTX and STR at 7 d (A) and 14 d (B) after MCAO. (C-D) Quantification of CD16+Iba1+ pro-inflammatory microglia/macrophages in the peri-infarct areas in CTX and STR at 7 d (C) and 14 d (D) after MCAO. n = 3–5 mice per group. *p < 0.05, **p < 0.01, ***p < 0.001. One-way ANOVA and Bonferroni post hoc tests. Data associated with this figure can be found in the supplemental data file (S1 Data). CTX, cortex; Iba1, ionized calcium binding adaptor molecule 1; IL-4, interleukin-4; MCAO, middle cerebral artery occlusion; STR, striatum. (TIF) [file pbio.3000330.s004.tif]

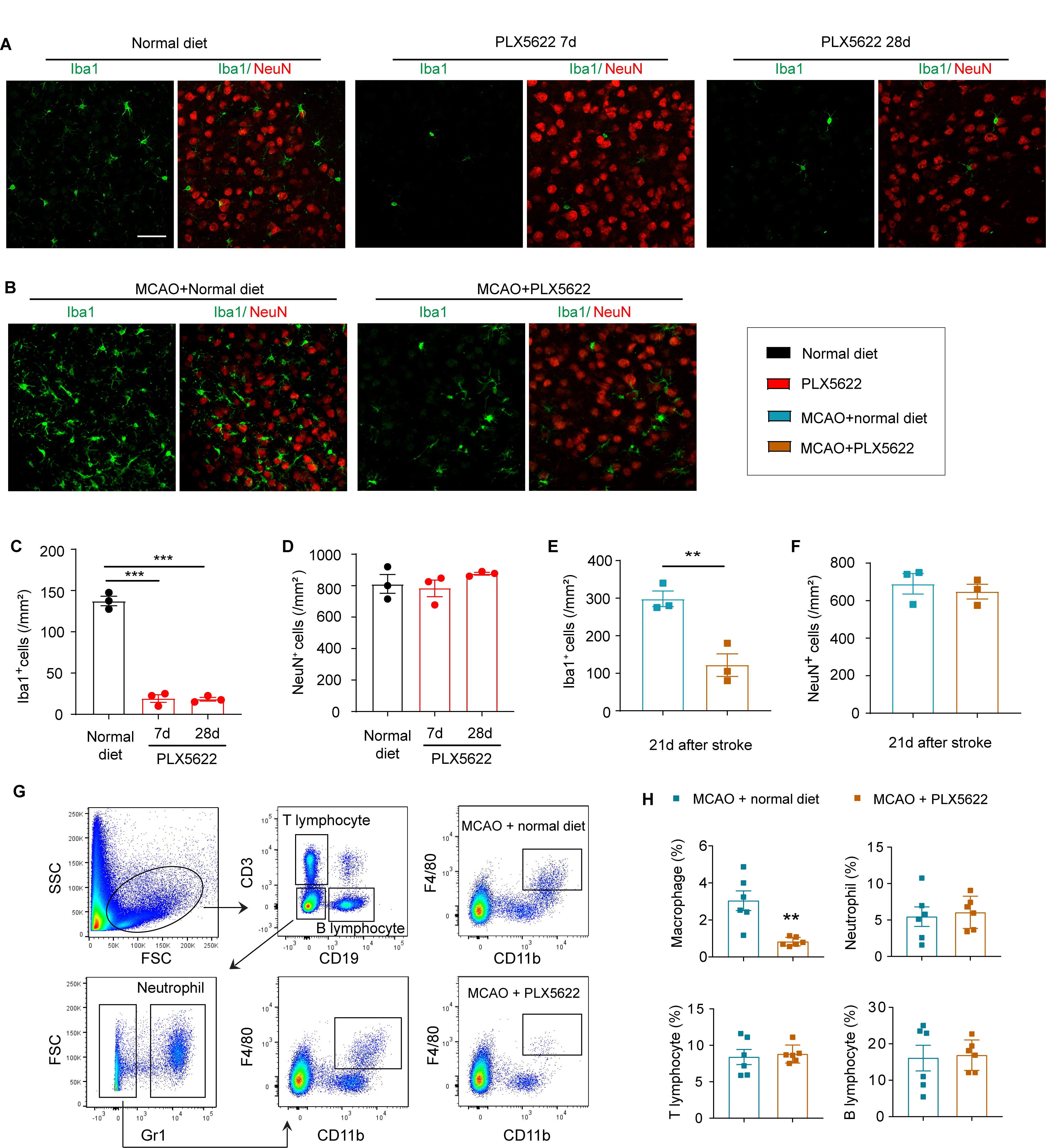

Supplement: S5 Fig — For microglia/macrophage depletion, PLX5622 was supplied in the diet (1,200 mg/kg of chow) to normal mice or stroke mice starting 7 d prior to 60-min MCAO and continued until sacrifice. (A-B) Representative images of double staining for Iba1 (green) and NeuN (red). Scale bar: 50 μm. (C-D) Quantification of Iba1+ microglia (C) and NeuN+ neurons (D) in sham brains 7 or 28 d after initiation of the PLX5622 diet. n = 3/group. (E-F) Quantification of Iba1+ microglia/macrophages (E) and NeuN+ neurons (F) in ischemic brains 28 d after initiation of the PLX5622 diet (21 d after stroke). n = 3/group. (G-H) Flow cytometry of the immune cells in the blood 28 d after initiation of the PLX5622 diet (21 d after stroke). Data are expressed as % of single cells. n = 6/group. **p < 0.01; ***p < 0.001. One-way ANOVA and Bonferroni post hoc test (C and D) or Student’s t test (E, F, and H). Data associated with this figure can be found in the supplemental data file (S1 Data). Iba1, ionized calcium binding adaptor molecule 1; MCAO, middle cerebral artery occlusion. (TIF) [file pbio.3000330.s005.tif]

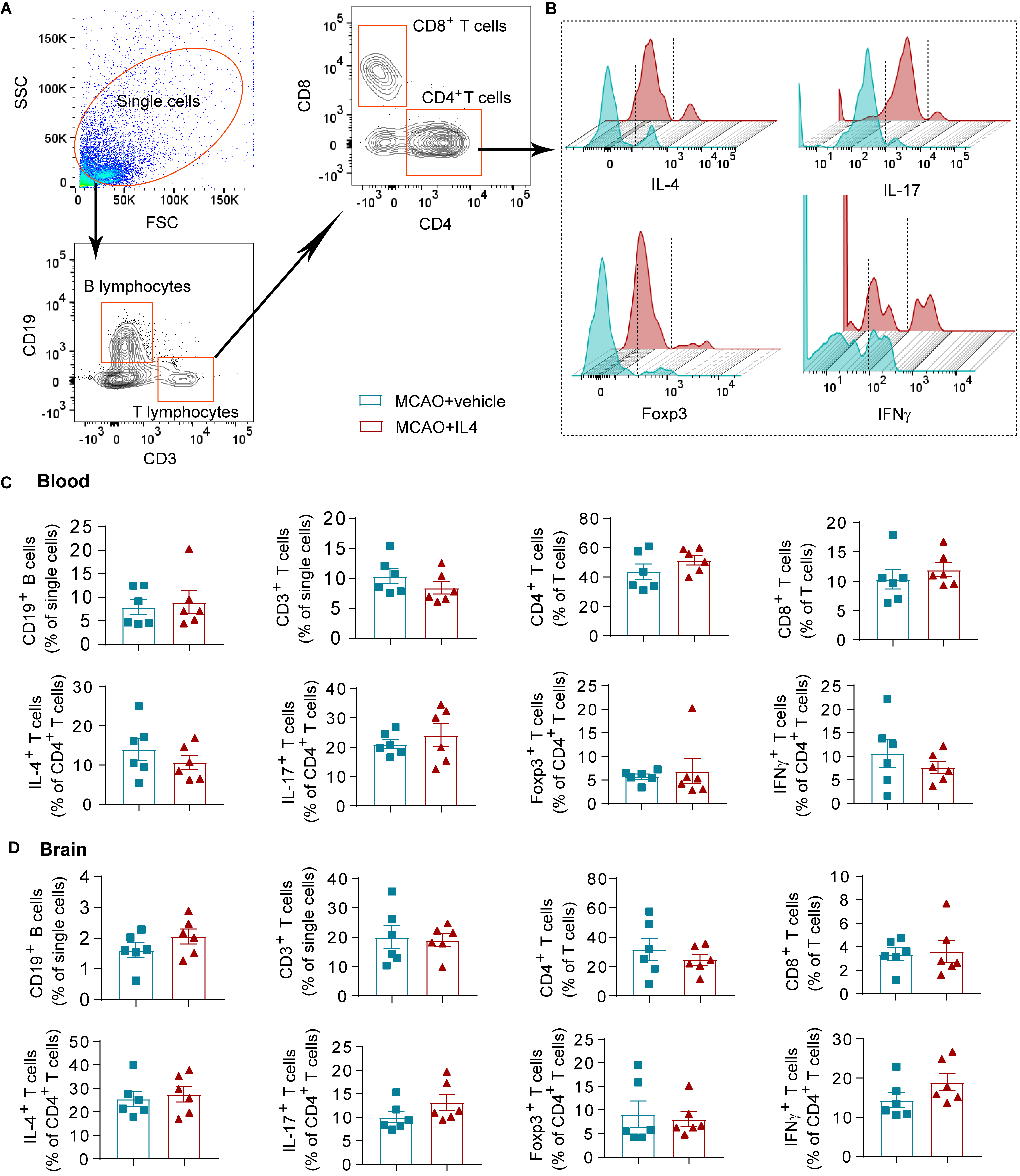

Supplement: S6 Fig — Transient focal ischemia was induced by 60-min MCAO. Stroke mice were post-treated with IL-4 or vehicle starting 6 h after MCAO at daily intervals for days 1–7. Animals were sacrificed on day 8 after stroke. (A) Gating strategy for blood lymphocyte populations. (B) Histograms showing the IL-4+ Th2 cell, IL-17+ Th17 cell, regulatory T cell (Foxp3+), and IFNγ+ Th cell subpopulations in CD4+ T cells in the blood of vehicle or IL-4-treated mice. (C) Quantification of lymphocyte populations in the blood. (D) Quantification of lymphocyte populations in ischemic brains. n = 6/group. Student’s t test. Data associated with this figure can be found in the supplemental data file (S1 Data). Foxp3, forkhead box P3; IFNγ, interferon gamma; IL-4, interleukin-4; MCAO, middle cerebral artery occlusion; Th, T helper. (TIF) [file pbio.3000330.s006.tif]

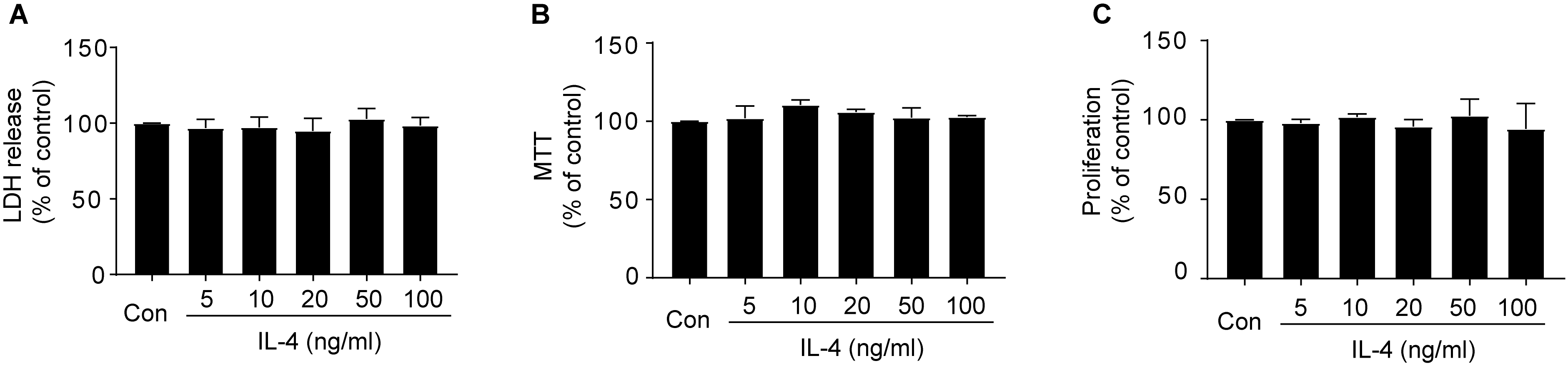

Supplement: S7 Fig — OPCs were treated with vehicle and various concentrations of IL-4 for 3 d. (A) LDH assay for cell death. (B) MTT assay. (C) Quantification of OPC proliferation using BrdU proliferation kit (Sigma-Aldrich). Three independent experiments, each performed in quadruplicate. Data associated with this figure can be found in the supplemental data file (S1 Data). BrdU, 5-bromo-2′-deoxyuridine; IL-4, interleukin-4; LDH, lactate dehydrogenase; OPC, oligodendrocyte progenitor cell. (TIF) [file pbio.3000330.s007.tif]

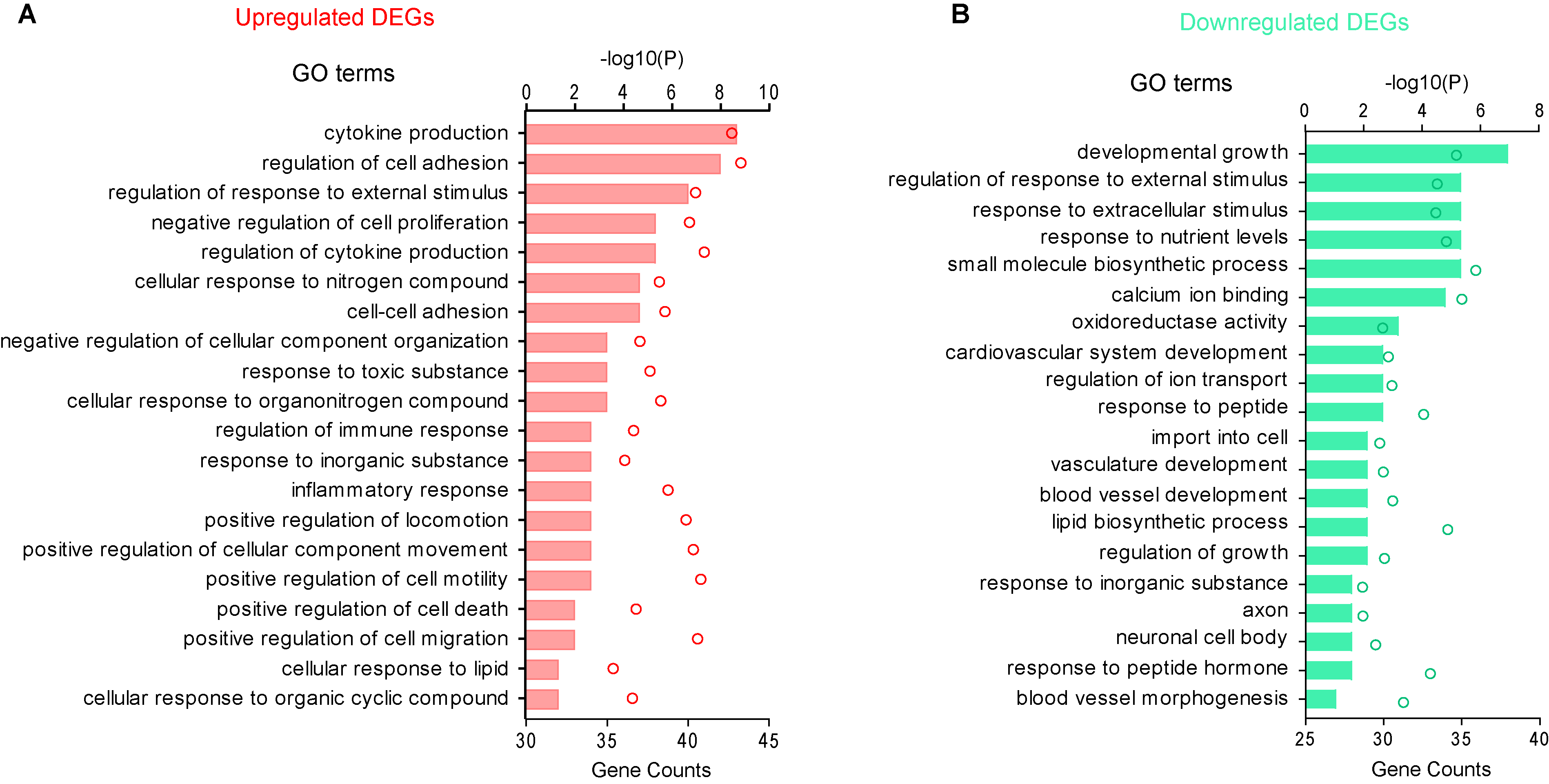

Supplement: S8 Fig — Microarray analyses were performed on OPCs treated with PBS or IL-4 (20 ng/mL) for 3 d. n = 4/group. DEGs that were up-regulated or down-regulated (FDR < 0.05; and fold change > 2) by IL-4 treatment were subjected to GO enrichment analysis using the Metascape analysis. The top 20 enriched GO terms in three categories (biological process, cellular component, and molecular function) are listed. Bars represent gene counts. Circles represent values of −logP. DEG, differentially expressed gene; FDR, false discovery rate; GO, gene ontology; OPC, oligodendrocyte progenitor cell. (TIF) [file pbio.3000330.s008.tif]

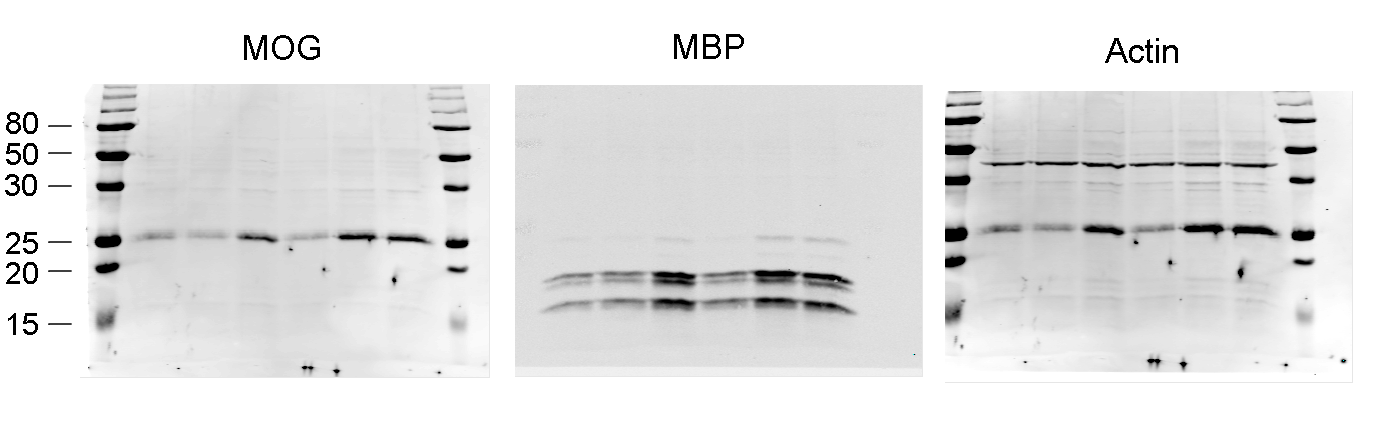

Supplement: S9 Fig — MBP, myelin basic protein; MOG, myelin oligodendrocyte glycoprotein. (TIF) [file pbio.3000330.s009.tif]

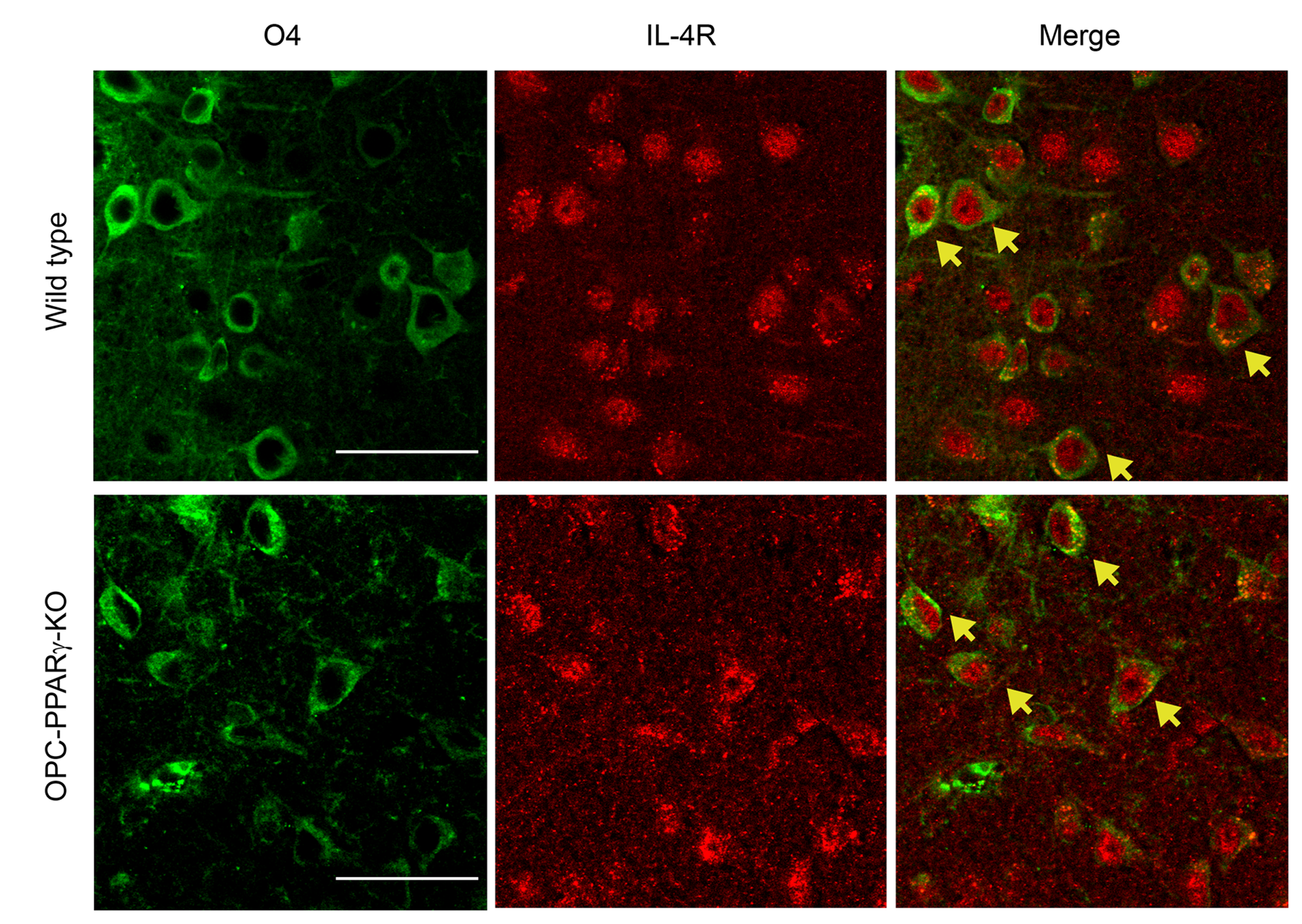

Supplement: S10 Fig — Brain slices were collected from WT or OPC-specific PPARγ KO mice 35 d after 60-min MCAO. Brain slices were stained for O4 (green)—a marker of preoligodendrocytes and premyelinating oligodendrocytes—and IL-4R (red). Yellow arrows indicate double-stained cells. Scale bar: 40 μm. IL-4R, interleukin-4 receptor; KO, knockout; MCAO, middle cerebral artery occlusion; OPC, oligodendrocyte progenitor cell; PPARγ, peroxisome proliferator-activated receptor gamma; WT, wild-type. (TIF) [file pbio.3000330.s010.tif]
